# Unraveling the Complexity of Regulated Cell Death in Esophageal Cancer: from Underlying Mechanisms to Targeted Therapeutics

**DOI:** 10.7150/ijbs.85753

**Published:** 2023-07-31

**Authors:** Haowen Zhang, Jin Zhang, Siyuan Luan, Zhiying Liu, Xiaokun Li, Bo Liu, Yong Yuan

**Affiliations:** 1 Department of Thoracic Surgery, West China Hospital, Sichuan University, Chengdu 610041, China.; 2 State Key Laboratory of Biotherapy and Cancer Center, West China Hospital, Sichuan University, Chengdu 610041, China.; 3 School of Pharmaceutical Sciences of Medical School, Shenzhen University, Shenzhen, 518000, China.

**Keywords:** Esophageal cancer, Cancer therapy, Regulated cell death, Small-molecule compounds, Long non-coding RNA

## Abstract

Esophageal cancer (EC) is the sixth most common and the seventh most deadly malignancy of the digestive tract, representing a major global health challenge. Despite the availability of multimodal therapeutic strategies, the existing EC treatments continue to yield unsatisfactory results due to their limited efficacy and severe side effects. Recently, knowledge of the subroutines and molecular mechanisms of regulated cell death (RCD) has progressed rapidly, enhancing the understanding of key pathways related to the occurrence, progression, and treatment of many types of tumors, including EC. In this context, the use of small-molecule compounds to target such RCD subroutines has emerged as a promising therapeutic strategy for patients with EC. Thus, in this review, we firstly discussed the risk factors and prevention of EC. We then outlined the established treatment regimens for patients with EC. Furthermore, we not only briefly summarized the mechanisms of five best studied subroutines of RCD related to EC, including apoptosis, ferroptosis, pyroptosis, necroptosis and autophagy, but also outlined the recent advances in the development of small-molecule compounds and long non-coding RNA (lncRNA) targeting the abovementioned RCD subroutines, which may serve as a new therapeutic strategy for patients with EC in the future.

## Introduction

Globally, esophageal cancer (EC) is regarded as a fatal gastrointestinal malignancy. According to the most recent global cancer statistics released by the International Agency for Research on Cancer (IARC), Esophageal Cancer (EC) ranks seventh in terms of overall cancer prevalence and sixth in terms of cancer-related mortality worldwide. In 2020, an estimated 604,000 new cases of EC were reported, resulting in approximately 544,000 deaths 1. EC can be classified into two broad histopathological subtypes: esophageal adenocarcinoma (EAC) and esophageal squamous cell carcinoma (ESCC). Globally, ESCC is the major subtype of EC, accounting for approximately 90% of all EC cases. Furthermore, while ESCC remains the main subtype of EC in eastern regions, South America and East Africa, the incidence of EAC has been on the rise in some developed western countries over the past decades, and EAC is now even the predominant subtype in Europe, the Americas and Australia 2,3. Owing to the absence of distinctive symptoms during the initial stages and the constraints of diagnostic techniques, individuals with EC frequently receive a diagnosis at an advanced and untreatable phase. 4. Depending on characteristics of the tumor (mainly the TNM stage) and patients (including fitness), different treatment options can be used to treat patients with EC, including endoscopic management, surgery, chemotherapy, radiotherapy and molecular targeted therapy. Despite significant advancements in medicine and cancer biology over the past decades, the existing EC treatments continue to yield unsatisfactory results due to their limited efficacy and severe side effects. The 5-year survival rate of EC remains less than 20%. Furthermore, decreased radiotherapy sensitivity and increased chemotherapy drug resistance of EC cells are becoming prominent, problems that urgently need to be solved. Therefore, the development of effective and rational therapies for patients with EC remains imperative 2,5,6.

Cell death, an inevitable destiny of all life, has physiological and pathological functions. According to functional characteristics, Cell death is commonly classified into two categories: regulated cell death (RCD) and accidental cell death (ACD). Unlike ACD, which is an uncontrolled process induced via unexpected attack and/or injury, RCD is closely related to precise signaling cascades involving a series of defined effector molecules 7. Kerr et al. described the concept of apoptosis and its role in physiological and pathological processes in the 1970s 8. Since then, RCD-related research has rapidly increased, and an increasing number of new nonapoptotic modes of RCD have been identified over the past few decades, including but not limited to autophagy, necroptosis, ferroptosis, pyroptosis, entosis, parthanatos, alkaliptosis, oxeiptosis, lysosome-dependent cell death, NETotic cell death and cuprotosis 9-11.

As one of the current research hotspots, dysregulation of single or mixed types of RCD subroutines has been linked to the development of various human diseases, especially cancer. The occurrence of malignant tumors significantly rises when there is a disruption in normal RCD or when abnormal cell proliferation is not properly controlled 12-15. For EC, accumulating evidence has shown that dysregulation of RCD subroutines can not only affect the occurrence and development of EC but also reduce its treatment effectiveness, for example, causing increased resistance to chemotherapy and immunotherapy and decreased radiotherapy sensitivity. Recently, researchers have devoted much effort to developing EC treatments that function by targeting RCD to promote individualized treatment of EC, decrease the risk of disease progression, improve the prognosis of patients and reduce toxicity. Using small-molecule compounds to target RCD subroutines has emerged as a promising therapeutic option for patients with EC, and rapid progress has been made in the past few years 16-19.

In this review, we firstly discussed the risk factors and prevention of EC. Subsequently, we outline established treatment regimens for patients with EC. In addition, we briefly summarize the mechanisms of five main types of RCD subroutines related to EC: apoptosis, autophagy, ferroptosis, pyroptosis and necroptosis. Importantly, we provide an overview of the latest progress in the advancement of small-molecule compounds that specifically target these five regulated cell death (RCD) subroutines. These compounds hold potential as innovative therapeutic approaches for individuals diagnosed with EC. Furthermore, we discuss the opportunities and challenges in this research field. We will provide the latest advances in the abovementioned RCD subroutine research and developments in the area of EC therapy. These findings will enhance the understanding of EC and provide some promising treatment options for patients with EC.

## Risk factors and prevention of EC

**Figure 1:**
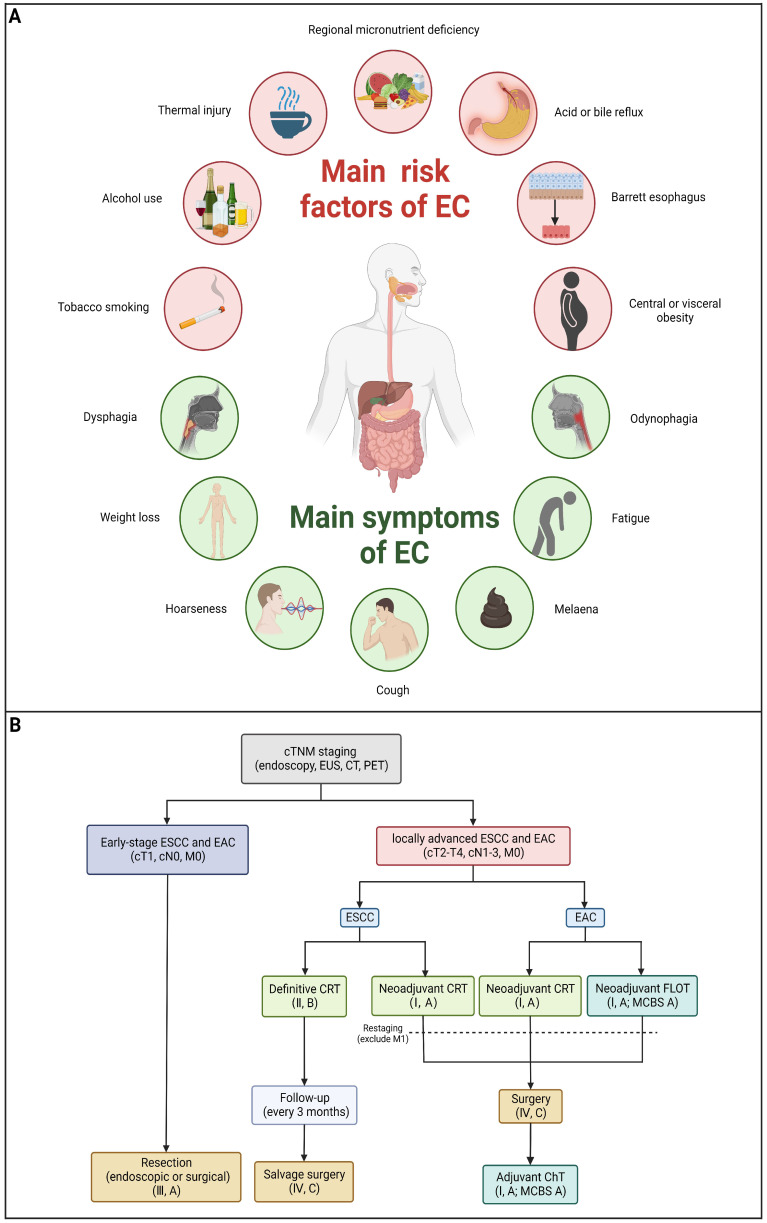
**Main risk factors, symptoms and current treatment algorithm of EC.** (A) Main risk factors and symptoms of EC. (B) Current treatment algorithm for patients with locally advanced EC. (Created with BioRender.com)

Risk factors differ between ESCC and EAC due to different mechanisms of carcinogenesis. Chronic irritation of carcinogens, mainly cigarette, alcohol and hot beverages, can increase the risk of the carcinogenesis of ESCC 20. Additionally, nutritional deficiencies are also found to increase the risk of ESCC 21. For EAC, the development of EAC is strongly linked to gastroesophageal reflux disease (GERD). Barrett's esophagus (BE) serves as a prominent risk factor for EAC 22. In addition, obesity was reported to be associated with a decreased risk of ESCC and an increased risk of EAC 23 **(Figure 1A)**.

In order to prevent EC, it is important to identify and eliminate risk factors mentioned above as the primary means of prevention. Furthermore, screening precancerous and early cancerous lesions in high-risk population is crucial to prevent, which can make curative treatment possible.

## Established treatment options for EC

To date, there are various methods to treat patients with EC, such as endoscopic resection, surgical resection, radiotherapy, chemotherapy, immunotherapy or some specific combination of these **(Table 1)**. Clinically, clinicians can decide the treatment for patients with EC based on both personal characteristics (such as fitness) and characteristics of the tumor, primarily its TNM stage 2,24,25. Patients with early-stage EC are treated primarily through surgery or endoscopic resection, and the therapeutic efficacy is astounding. In terminal cases, surgery is not sufficient and is often combined with preoperative or perioperative chemotherapy/chemoradiotherapy. Although the treatment options for patients with advanced EC are constantly being optimized, the poor quality of life and poor five-year survival rate are still disappointing. In addition, increased resistance to chemotherapy and immunotherapy and decreased radiotherapy sensitivity of EC cells are becoming prominent, problems that urgently need to be solved. Below, we summarize the current available treatment strategies for EC. As shown in **Figure 1B**, an algorithm has been formulated to address the treatment of individuals diagnosed with locally advanced EC.

### Endoscopic resection

Endoscopic resection has emerged as a treatment option for lesions with intraepithelial high-grade dysplasia as well as most T1 tumors. The indication for ER depends on lymph node metastasis risk. The development of lymph node metastases rarely occurs among lesions in which the depth of invasion does not extend beyond the mucosal layer (T1a); therefore, ER can be curative in these cases. ER is also an option if lesions extend into the muscularis mucosae or slightly infiltrate the submucosa (up to 200 mm), which are regarded as relative indications. At present, ER used for early EC patients can typically be categorized into cap-assisted endoscopic mucosal resection (EMRC), ligation-assisted endoscopic mucosal resection (EMRL), multiband mucosectomy (MBM) and endoscopic submucosal dissection (ESD) 25-27.

### Surgery

Surgery is often required for nonmetastatic, locally advanced ESCC and EAC (stages T1b-T4, N1-N3, M0). Currently, various methods for EC resection based on different approaches and the extent of lymphadenectomy are used, such as McKeown esophagectomy, Ivor Lewis esophagectomy and Sweet esophagectomy 2,28,29. Furthermore, in recent years, minimally invasive esophagectomy (MIO) techniques such as robotics techniques have become more common clinically and are reported to have quicker functional recovery, lower postoperative morbidity and improved quality of life (QoL) 25,30,31.

### Preoperative and perioperative treatment

According to the latest ESMO Clinical Practice Guideline, all cases with locally advanced resectable EC should be carefully considered for preoperative or perioperative ChT/CRT, which are expected to reduce the primary tumor volume, increase rates of radical (R0) resection and survival and decrease the recurrence risk. Moreover, preoperative treatment using ChT or CRT can not only attenuate dysphagia and improve nutritional status among most patients but also reduce the requirement for feeding tube placement. Furthermore, it is worth mentioning that the need for preoperative treatment of cT2 N0 tumors is currently controversial, and further study is required 25,32,33.

### Definitive CRT

According to the latest ESMO Clinical Practice Guideline, definitive CRT (with salvage surgery and close surveillance) is a recommended treatment for patients with resectable EC. Furthermore, for patients with EC who are unwilling or unable to undergo surgery, definitive CRT should also be considered 25,34.

### Palliative care

In recent years, palliative care has increasingly played a vital role in EC patients with numerous and complex symptoms caused by tumor location and required treatments, such as dysphagia, malnutrition, pain and psychological symptoms 35. Palliative chemotherapy is initiated in patients with advanced EC who experience dysphagia and malnutrition caused by esophageal stenosis and aspiration induced by a fistula, with the goal of improving their symptom burden and QoL. Various approaches have been developed for the palliation of advanced EC, such as self-expanding metal stents (SEMSs), stents loaded with (125) iodine seeds, brachytherapy and external radiation therapy 36-38.

## RCD subroutines and EC treatment

Despite the availability of multimodal therapeutic strategies at present, the five-year survival rate of patients with EC is consistently less than 20%. In addition, increased resistance to chemotherapy and immunotherapy as well as decreased radiotherapy sensitivity of EC cells is becoming prominent. Thus, there is a pressing need to optimize existing clinical treatments for patients with EC and to find novel and effective treatment options. In this context, accumulating evidence has shown that using small-molecule compounds to precisely target specific RCD patterns is a promising approach to treat patients with EC, which is expected to improve the clinical outcomes of existing treatment modalities, including chemotherapy, radiotherapy and immunotherapy. Here, we briefly summarize the mechanisms of five main types of RCD subroutines that are associated with EC and review recent advances in the development of RCD-related small-molecule compounds in the field of EC.

## Targeting apoptotic pathways with small-molecule compounds in EC

### Brief introduction to apoptosis

**Figure 2:**
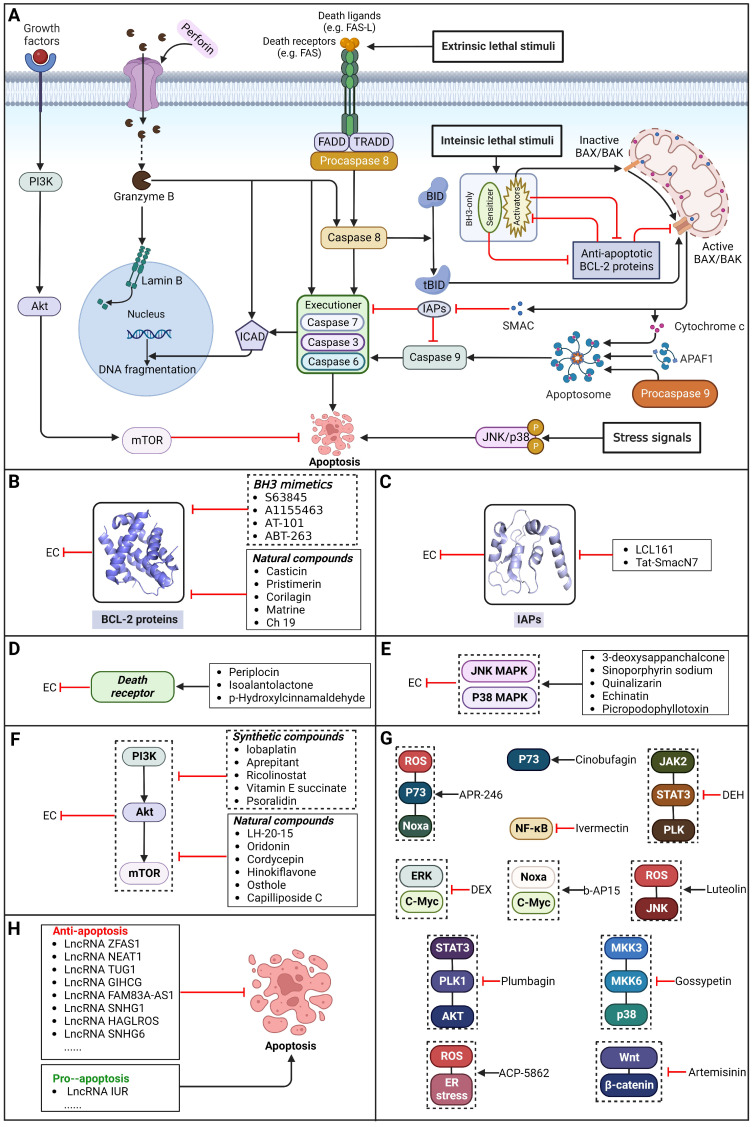
** Core apoptotic pathways in EC and EC therapeutic approaches by targeting apoptosis pathways.** (A) Core apoptotic pathways in EC. Apoptosis is a form of typical RCD characterized by a series of morphological changes and the formation of so-called apoptotic bodies and is mainly activated via the intrinsic pathway, the extrinsic pathway and the granzyme-perforin pathway. (B) Small-molecule compounds targeting apoptosis-related BCL-2 family proteins in EC. (C) Small-molecule compounds targeting apoptosis-related IAP proteins in EC. (D) Small-molecule compounds targeting apoptosis-related death receptor in EC. (E) Small-molecule compounds targeting apoptosis-related the JNK/p38 MAPK pathway in EC. (F) Small-molecule compounds targeting apoptosis-related the PI3K/AKT/mTOR pathway in EC. (G) Small-molecule compounds targeting apoptosis-related other regulators in EC. (H) Some lncRNAs targeting apoptosis-related pathways in EC. (Created with BioRender.com)

Apoptosis, a conventional type of regulated cell death (RCD), is distinguished by a sequence of morphological alterations and the generation of apoptotic bodies (ABs). It is predominantly triggered through the intrinsic pathway (also referred to as the mitochondrial pathway) and the extrinsic pathway (also known as the death receptor pathway) 10. Additionally, apoptosis can also be induced by perforin and granzyme B released from immune cells, which is called the granzyme-perforin pathway 39. In general, the intrinsic pathway can be activated via various intracellular stress signals, such as ionizing radiation, chemotherapy, targeted therapy, endoplasmic reticulum stress and growth factor deprivation 10. The extrinsic pathway can be activated via interactions between members of the tumor necrosis factor receptor (TNFR) superfamily and their corresponding ligands (e.g., FAS can be activated by FAS ligand) 40 **(Figure 2)**.

### Targeting BCL-2 family proteins

During apoptosis, B-cell lymphoma-2 (BCL-2) family proteins act as central regulators. The intrinsic pathway of apoptosis is controlled tightly through balance between BCL-2 protein family members, including anti-apoptotic BCL-2 proteins (e.g., BCL-XL, BCL-2 and MCL-1), pro-apoptotic BH3-only proteins and pro-apoptotic effector proteins (e.g., BAX and BAK1). Normally, by binding and sequestering proapoptotic BH3-only 'activator' proteins and effector proteins, BCL-2 proteins can act against apoptosis 41. BH3-only proteins can be upregulated to promote apoptosis in response to intracellular stress signals. Therefore, patients with EC may benefit from selectively targeting BCL-2 family proteins to induce apoptosis.

#### BH3 mimetics

Notably, BH3 mimetics are regarded as a novel class of antitumor agents that can directly target BCL-2 family proteins and induce MOMP in a BAK/BAX-dependent manner 42,43 and show certain potential in the treatment of EC. Researchers have tested BH3-mimetic drugs in preclinical cancer models, and some drugs that target MCL-1 and BCL-XL have been assessed in phase I clinical trials for certain cancers 44. It is worth mentioning that the BCL-2-specific inhibitor **venetoclax** is approved by many regulatory authorities worldwide, including the US Food and Drug Administration, to treat acute myeloid leukemia and chronic lymphocytic leukemia, and there is potential for an expanding range of indications 45. For EC, BCL-XL and MCL-1 were found to be highly expressed in EAC cell lines and tumor samples. The BH3 mimetics **S63845** and **A1155463** were further confirmed to be able to activate the BAX/BAK-dependent mitochondrial apoptotic pathway and sensitize EAC to chemotherapeutics by targeting MCL-1 and BCL-XL respectively 46. In addition, a natural BH3-mimetic molecule named **AT-101** has been reported to decrease the expression of MCL-1 and BCL-2, leading to apoptosis of EAC cells, and thus has a favorable anti-EC and clinical effect 47,48. Moreover, a novel BH3 mimetic named **ABT-263** with high affinity for BCL-XL, BCL-2 and BCL-w has been proven to exert both cytotoxic and cytostatic effects on EC cells *in vitro* and has entered clinical trials for the treatment of cancer. **ABT-263** dose-dependently suppressed the viability of 3 EC cell lines with IC_50_ values of 8.2±1.6, 7.1±1.5 and 10.7±1.4 μmol/L in CaES-17, HKESC-2 and EC109 cells, respectively 49. Hopefully, using BH3-mimetic drugs alone or in combination with other drugs has broad prospects in clinical application **(Table 2)**.

#### Natural compounds

To regulate apoptosis, natural compounds and their derivatives have gradually become a new source of inspiration and have the advantages of easy preparation, low multidrug resistance and low cytotoxicity. Some of these natural compounds have been proven to exert anti-EC activity by targeting BCL-2 family proteins. For instance, a flavonoid named **casticin** has been reported to induce apoptosis of ESCC cells by repressing BCL-2 expression and upregulating BAX, cleaved caspase-3, caspase-9, and cleaved PARP, as well as activating the JNK signaling pathway 50. Moreover**,** a natural quinonoid triterpene named **pristimerin** was found to downregulate BCL-2 expression and upregulate the expression of BAX and caspase-3 and caspase-9, resulting in apoptosis of ESCC cells. Further results of *in vivo* experiments demonstrated that **pristimerin** also inhibited tumor growth in an EC mouse model 51. In addition, a tannin named **corilagin** was found to promote apoptosis of ESCC Eca109 and KYSE150 cells by increasing the BAX/BCL-2 ratio as well as the expression of cleaved caspase-3, caspase-8, and caspase-9^52^. Moreover, the Chinese traditional herbal medicine **matrine** induces apoptosis of ESCC cells by downregulating the expression of BCL-2 and upregulating the expression of BAX and caspase-3, caspase-8 and caspase-9, as well as increasing ROS 53. In addition, **2,4,6‑trimethoxy‑4'‑nitrochalcone (Ch‑19)**, a new synthetic chalcone derivative, has been reported to exert an anti-EC effect by inducing ROS accumulation in and apoptosis of EC cells. In ESCC Eca109 and KYSE450 cells, **Ch-19** promoted apoptosis by downregulating BCL-2 expression, upregulating Bad, Bim, PUMA and BAX expression, and activating PARP and caspase-3 54 **(Table 2)**.

Overexpression of BCL-2 family proteins renders cancer cells resistant to apoptotic signals, accelerating cancer progression and reducing treatment response. Thus, using synthetic chemical compounds or natural product-derived compounds to inhibit BCL-2 family proteins has critical importance in the treatment and prognosis of EC 55. However, these compounds have ongoing crucial challenges for clinical application, which are in large part due to their on-target effects on normal tissues and organs. In addition, the poor stability and short half-life of some of the compounds mentioned above are also major issues that have seriously impaired their clinical application. These challenges can potentially be overcome by utilizing delivery systems based on stable, efficient and highly targeted vectors, such as exosomes derived from human tumor cells or normal cells. Consequently, targeting BCL-2 family proteins to induce apoptosis has great potential to treat patients with EC, and further study of delivery systems combined with other agents, such as exosome-based carriers combined with various BCL-2 inhibitory preparations, have much promise for EC treatment.

### Targeting IAP proteins

As anti-apoptotic proteins, IAPs are reported to be overexpressed in various cancers, including EC, and to be linked to resistance to chemotherapy. Therefore, targeting IAPs, including XIAP, cIAP1, and cIAP2, is generally considered a promising therapeutic strategy 56-58. The endogenous mitochondrial protein Smac can promote apoptosis by antagonizing IAP proteins. Researchers found that the expression of Smac in EC cell lines and in tumor specimens from patients with EC was downregulated, which was believed to be related to decreased sensitivity to EC chemotherapy 59. Smac mimetics, synthetic small-molecule peptides, have been designed and developed as a new class of targeted drugs that can mimic the proapoptotic mitochondrial protein Smac for cancer treatment in recent years 60. In general, Smac mimetics can be divided into two types: monovalent and bivalent, which contain one and two SMAC-mimicking units, respectively. To date, Smac mimetics, including but not limited to five monovalent compounds (**CUDC-427/GDC-0917**, **Debio 1143/AT-406/SM-406**, **LCL161, ASTX660** and **RG7419/GDC-0152**) and three bivalent agents (**AEG40826/HGS1029**, **APG-1387/SM-1387** and** birinapant/TL32711**), have been evaluated as therapeutic agents for solid tumors and hematological cancers in early clinical trials. In these clinical trials, Smac mimetics were used alone or in combination with other treatments and demonstrated clinical efficacy and acceptable safety 61. The Smac mimetic **LCL161** was reported to enhance apoptosis by downregulating the expression of cIAP1 and promoting the activation of caspase 8, which acts as a strong radiosensitizer to X-ray irradiation in ESCC cells 62. Similarly, another study also reported that **LCL161** could promote apoptosis of Eca109 cells in a dose-dependent manner by removing the repression of caspase by XIAP 63. Furthermore, a Smac mimetic named **Tat-SmacN7** was developed and shown to be a new and potent radiosensitizer of human EC cells because it induces apoptosis mediated by caspase activation by removing the negative blockers XIAP and cIAP-1 64. In summary, these findings indicate that using Smac mimetics alone or in combination with conventional anticancer drugs can produce better effects for the treatment of EC. Further studies of Smac mimetics may help develop novel therapeutic strategies for patients with EC **(Table 2)**.

### Targeting death receptor-induced apoptosis

Among TNFR superfamily members, DR4 and DR5 are significantly increased in cancer cells compared to normal cells and thus are regarded as the most promising candidates for targeted therapy for tumors. In humans, interaction between TNF-related apoptosis-inducing ligand (TRAIL) and DR4 or DR5 can lead to the assembly of DISC, which is followed by caspase-8-dependent apoptosis. Consequently, recombinant human TRAIL (RhTRAIL) protein or agonistic antibodies targeting DR4 and DR5, also known as proapoptotic receptor agonists (PARAs), have been developed and found to induce apoptosis of several different cancer cell lines 65. Additionally, numerous studies have shown that PARAs and rhTRAIL can enhance the sensitivity of tumors to therapies, including radiotherapy, chemotherapy, and targeted therapy 66-69. Nevertheless, clinical trials of TRAIL have not demonstrated satisfactory results, which is thought to be due to acquired resistance of tumor cells to TRAIL-induced apoptosis 70. Researchers have thus shown a keen interest in compounds that are capable of increasing the expression of death receptors. Compounds that can sensitize cells to TRAIL-induced apoptosis can be divided into three groups: 1) clinically used anticancer drugs (e.g., **etoposide**, **mitomycin c** and **mitoxantrone**) 71-73, 2) natural compounds with anticancer properties (e.g., **ginsenoside compound K**, **magnolol, polyphenol mixture** and **chikusetsusaponin IVa butyl ester**) 74-76, and 3) anticancer agents that are currently being developed (e.g., **Nutlin-3**, **capsazepin** and **GW280264X**) 77-79. For EC, a natural compound named **periplocin (CPP)** alone or in combination with TRAIL was found to dose-dependently increase the expression of DR4/DR5, FADD and cleaved caspase-3 by inhibiting FoxP3, inducing apoptosis of ESCC cells and making ESCC cells more susceptible to TRAIL-induced apoptosis 80. Similarly, a major sesquiterpene lactone named **isoalantolactone** was reported to promote apoptosis by upregulating DR5 and ROS in human EC cells 81. In addition, **p-hydroxylcinnamaldehyde (CMSP)** is an extract from traditional Chinese medicine that has been proven to enhance TRAIL-induced apoptosis of ESCC cells by increasing the expression of DR4 and DR5 and activating the p38 MAPK signaling pathway 82. These sensitizers alone or in combination with a TRAIL-related agent might be novel clinical treatment options for patients with EC **(Table 2)**.

### Targeting the JNK/p38 MAPK pathway

Mitogen-activated protein kinase (MAPK) signaling pathways have crucial roles in various cellular networks related to the cell cycle, cell growth, cell survival and cell death 83. Activation of MAPK pathways by ROS has been linked to induction of cell apoptosis. By sequential phosphorylation of MAPK, it is possible to activate members of the MAPK pathway, such as JNK and p38. Activating p38 in the MAPK signaling pathway can promote apoptosis of tumor cells and halt tumor formation 84. JNKs can regulate the apoptotic signaling pathway by either regulating the activities of proapoptotic or antiapoptotic proteins or inducing the expression of apoptotic genes 85. Therefore, patients with EC may benefit from selective targeting of the JNK/p38 MAPK pathway. A recent study reported that a chemical separated from Caesalpinia sappan L named **3-deoxysappanchalcone (3-DSC)** can induce cell cycle arrest and ROS-mediated apoptosis of EC cells via the JNK/p38 MAPK signaling pathway 86. Similarly, **sinoporphyrin sodium (DVDMS)**, a novel sensitizer isolated from photofrin, can induce ROS-mediated apoptosis in combination with photodynamic therapy (PDT) via the JNK/p38 MAPK signaling pathway 87. In addition, **quinalizarin (Quina)**, a prominent constituent of several herbal medicines, is believed to exert antitumor effects in EC, which can induce apoptosis by modulating MAPK, STAT3, and NF-κB signaling pathways through the generation of ROS 88. A retrochalone from licorice, **echinatin (Ech)**, was found to promote apoptosis of ESCC cells by generating ROS/ER stress as well as activating the JNK/p38 MAPK signaling pathway 89. Moreover, as an epimer of podophyllotoxin isolated from the roots of Podophyllum hexandrum, **picropodophyllotoxin (PPT)** has been reported to exert antitumor effects by inducing apoptosis via upregulation of ROS levels and activation of the JNK/p38 signaling pathways 90 **(Table 2)**. Thus, it might be possible to treat EC by regulating JNK/p38 MAPK pathway-mediated apoptosis.

### Targeting the PI3K/AKT/mTOR pathway

The phosphatidylinositol 3-kinase (PI3K)/protein kinase B (AKT)/mammalian target of rapamycin (mTOR) pathway regulates some common cellular functions that are also crucial for tumorigenesis, such as cell metabolism, angiogenesis, cell proliferation, cell cycle progression and apoptosis 91.

#### Synthetic compounds

As a third-generation platinum antineoplastic drug, **lobaplatin (LBP)** can effectively induce apoptosis, repress proliferation and enhance radiosensitivity by inhibiting the PI3K/AKT pathway in ESCC 92. Furthermore, a nonpeptide NK1R antagonist named **aprepitant** has been reported to induce apoptosis and G2/M arrest via the PI3K/Akt/NF-κB axis in ESCC spheres 93. Furthermore, **ricolinostat (ACY-1215)**, a selective HDAC6 inhibitor, has been shown to induce apoptosis and G2/M arrest in ESCC cells through the PI3K/AKT/mTOR and ERK pathways, which were then confirmed *in vivo* 94. As a potential chemical agent for cancer prevention and therapy, **vitamin E succinate (VES)** was found to induce apoptosis by blocking the PI3K/AKT/mTOR axis in ESCC cells 95. Moreover, **psoralidin** could effectively inhibit proliferation and enhance apoptosis by inhibiting the PI3K/Akt and NF-κB signaling pathways in ESCC cells 96 **(Table 2)**.

#### Natural compounds

The use of natural compounds for the treatment of human diseases has a long history, and these compounds are thus an important resource for developing drugs. For EC, **LH-20-15**, a cytotoxic extract obtained from Gekko japonicus, demonstrates the ability to inhibit the proliferation and induce apoptosis of ESCC cells, which are achieved through the inhibition of the PI3K/Akt/GLUT1 signaling pathway 97. In addition, as an active diterpenoid isolated from Rabdosia rubescens, **oridonin** was also found to cause mitochondria-dependent apoptosis of ESCC cells by inhibiting the Ras/Raf and PI3K/AKT/mTOR pathways. An *in vivo* experiment demonstrated that treatment with oridonin inhibited tumor growth in an EC mouse model 98. **Cordycepin**, a nucleoside analog, has been shown to augment the chemosensitivity of ESCC cells to cisplatin. This effect is achieved by inhibiting the PI3K/AKT/mTOR signaling pathway and activating AMPK, representing a novel potential treatment for EC 99. In addition, **hinokiflavone**, a natural biflavonoid compound, has been reported to induce the apoptosis and inhibit the proliferation of ESCC cells by blocking the PI3K/AKT/mTOR signaling pathway 100. In addition, an active component extracted from the fruit of Fructus cnidii named **osthole** promoted cell cycle arrest in and the apoptosis of ESCC cells by inhibiting the PI3K/AKT signaling pathway 101. **Capilliposide C (CPS-C)** is an extract of a traditional Chinese medicine that can sensitize ESCC cells to chemotherapy by promoting apoptosis by inhibiting the PI3K/Akt/mTOR pathway 102. In summary, regulating apoptosis by targeting the PI3K/AKT/mTOR axis is a novel and promising therapeutic strategy for patients with EC** (Table 2)**.

### Targeting other apoptosis regulators

New targets for the treatment of EC have also been studied in recent years along with the above common targets. For instance, d**exmedetomidine (DEX)** has been shown to suppress the proliferation and induce apoptosis of EC cells by inhibiting the expression of the C-Myc gene through the blockade of the ERK signaling pathway 103. In addition, in a recent library screening using cells harboring mutant p53, the small-molecule compound** APR-246** was identified**.** Further study demonstrated that **APR-246** induced pronounced antitumor effects by promoting ROS-p73-Noxa-mediated apoptosis of ESCC cell lines bearing p53 missense mutations. These observations were confirmed using xenografts and patient-derived xenograft (PDX) models of p53-mutant ESCC 104. Moreover,* in vitro* and *in vivo* experiments were performed to confirm that combining **luteolin** with low-dose **paclitaxel** promotes ROS/JNK pathway-mediated apoptosis of ESCC cells 105. Additionally, a natural quinoid constituent named **plumbagin** was found to inhibit the proliferation and promote the apoptosis of ESCC cells *in vivo* and *in vitro* by inhibiting STAT3-PLK1-AKT signaling 106. In addition, as a hexahydroxylated flavonoid found in many flowers and hibiscus, **gossypetin** was found to induce apoptosis of ESCC cells *in vivo* by inhibiting the MKK3/MKK6/p38 signaling pathway, which might prevent the development of EC among healthy individuals, especially those who are at high risk of developing this cancer 107. **Artemisinin (ART)**, an antimalarial compound, has been reported to exhibit inhibitory effects on cell proliferation, migration, and invasion in addition to inducing apoptosis of ESCC cells. These effects are attributed to the repression of the Wnt/β-catenin signaling pathway 108. Additionally, a polycyclic lactone pesticide produced by Streptomyces avermitilis named **ivermectin** was found to promote apoptosis of ESCC cells by increasing ROS accumulation and repressing the NF-κB signaling pathway 109. A recent study demonstrated that one of the sesquiterpene lactones, **dehydrocostus lactone (DEH),** could induce cell cycle arrest in and promote the apoptosis of ESCC cells by repressing the JAK2/STAT3/PLK signaling pathway, inducing a potent antitumor effect 110. Furthermore, the traditional Chinese herbal extract **cinobufagin** has been reported to promote cell cycle arrest and apoptosis to block the growth of ESCC cells by activating the p73 signaling pathway 111. The deubiquitinase inhibitor **b-AP15** has been identified as capable of inducing apoptosis in ESCC cells by upregulating c-Myc and Noxa. These findings suggest its potential utility as a therapeutic agent for ESCC 112. Moreover, as a major metabolite of acalabrutinib, **ACP-5862** has been reported to exert antitumor effects in ESCC by promoting ER stress-mediated apoptosis by upregulating the production of ROS 113 **(Table 2)**. These findings provide additional potential novel therapeutic apoptosis-related targets and pathways for treating EC that may have far-reaching ramifications in the future.

### LncRNAs regulating apoptosis in EC

Notably, lncRNAs, which are endogenous cellular RNAs longer than 200 nucleotides and do not encode proteins, have been shown to play a significant role in the initiation and progression of several cancers, including EC. For instance, **lncRNA SNHG7** was significantly upregulated in EC cells and tissues, and SNHG7 was found to enhance ESCC cell proliferation and inhibit ESCC cell apoptosis through the modulation of p15 and p16 expression 114. In addition, downregulation of **lncRNA IUR** has been reported in ESCC, and it is associated with an unfavorable prognosis in ESCC patients. Further study proved that IUR could regulate cancer cell proliferation and the apoptosis of ESCC cells via miR‑21 and PTEN 115. Moreover, **lncRNA ZFAS1** was found to be upregulated in ESCC tissues, and further study proved that ZFAS1 could activate the proliferation, migration and invasion of ESCC cells and suppress their apoptosis by regulating miR-124 and STAT3 116. In addition to the lncRNAs mentioned above, other lncRNAs that can regulate the apoptosis of EC cells are also summarized in **Table 6** 117-138. LncRNAs may be useful biomarkers for diagnosing EC and predicting prognosis and relapse, and the ongoing development of drugs targeting these lncRNAs might produce new therapeutic targets.

Developing drugs that target apoptotic pathways directly has great potential to cause tumor regression in difficult-to-treat EC, and thus, these drugs are expected to be used in combination with other treatments, such as immunotherapy, drugs targeting oncogenic survival pathways, radiotherapy, and chemotherapy. Furthermore, lncRNAs may be useful in the diagnosis and prognostication of EC and even be drug targets. Undoubtedly, targeting apoptotic pathways with small-molecule compounds is a promising method for EC therapy, warranting further in-depth investigations.

## Targeting autophagy pathways with small-molecule compounds in EC

### Brief introduction to autophagy

**Figure 3:**
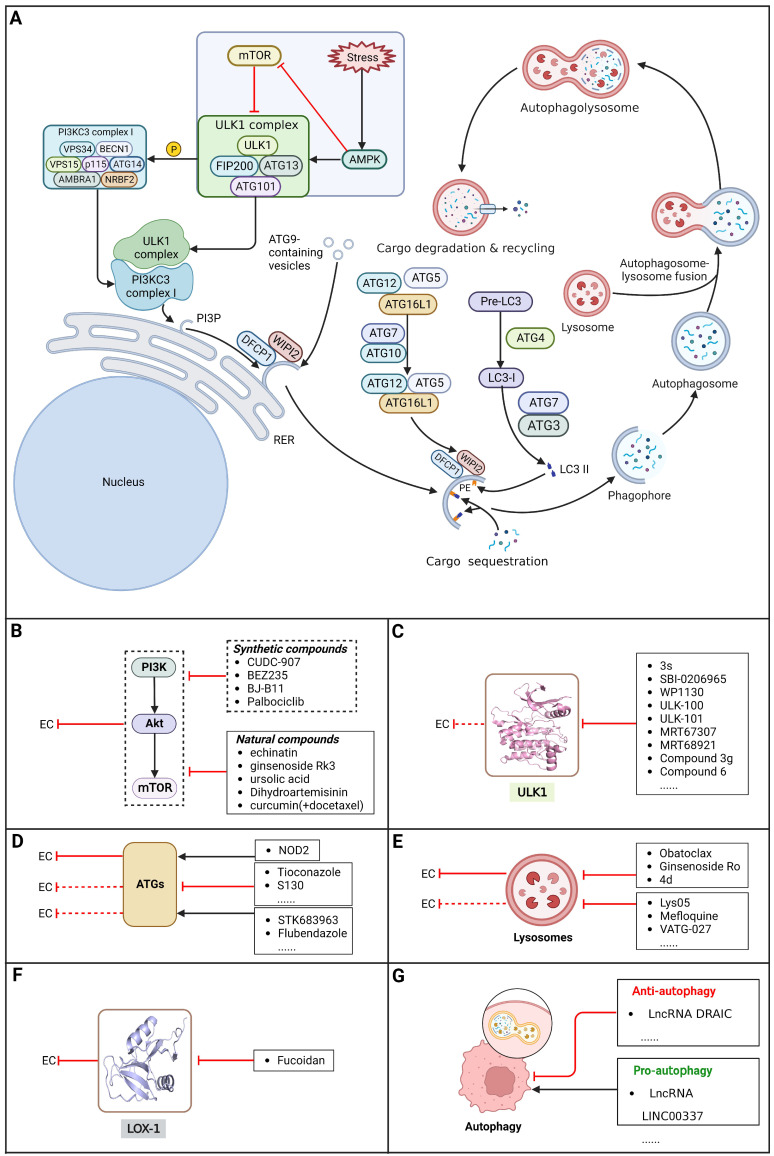
** Core autophagy pathways in EC and EC therapeutic approaches by targeting autophagy pathways.** (A) Core autophagy pathways in EC. The autophagy process can be conceptualized as consisting of five distinct stages: 1) initiation, 2) nucleation of the phagophore, 3) formation of the autophagosome, 4) fusion of the autophagosome and lysosome, and 5) cargo degradation and recycling. (B) Small-molecule compounds targeting autophagy-related the PI3K/AKT/mTOR pathway in EC. (C) Small-molecule compounds targeting autophagy-related ULK1 in other cancers. (D) Small-molecule compounds targeting autophagy-related ATG proteins in EC and other cancers. (E) Small-molecule compounds targeting autophagy-related lysosomes in EC and other cancers. (F) Small-molecule compounds targeting autophagy-related LOX-1 in EC. (G) Some lncRNAs targeting autophagy-related pathways in EC. (Created with BioRender.com)

Autophagy, first coined by Christian de Duve in 1963, refers to an evolutionarily conserved intracellular catabolic process involving the formation of double-membraned vesicles named autophagosomes that causes cellular components (e.g., organelles, proteins and lipids) to be delivered to lysosomes for degradation and recycling, contributing to the maintenance of cellular homeostasis. The autophagy process can be conceptualized as consisting of five distinct stages: 1) initiation, 2) nucleation of the phagophore, 3) formation of the autophagosome, 4) fusion of the autophagosome and lysosome, and 5) cargo degradation and recycling **(Figure 3)** 139-141. As a multistep lysosomal degradation process, autophagy has a dynamic role in the progression of cancer based on the context. In the earliest stages of tumorigenesis, autophagy can serve as a suppressor of tumor growth by eliminating mutated proteins and oncogenes that possess the ability to induce cellular mutations and/or cancer. Nevertheless, as the tumor advances to an advanced stage, autophagy can transition into a defensive and survival mechanism for cancer cells. It contributes to the survival, growth, and metastasis of established tumors by providing essential nutrients to cope with environmental stresses, including hypoxia, DNA damage, metabolic stress, nutrient shortage and cancer therapy 142,143. In this context, determining how to take advantage of this "double-edged sword" in the treatment of EC via external interventions such as small-molecule compounds have been a hot research topic among cancer researchers.

### Targeting the PI3K/AKT/mTOR pathway

Besides its role in regulating apoptosis, the PI3K/Akt/mTOR pathway is reported as the central pathway regulating autophagy and is regarded as a crucial survival pathway in tumor cells related to aggressive growth and malignant progression. Mounting evidence supports the ability of small-molecule compounds to induce autophagy in EC cells through modulation of the PI3K/AKT/mTOR pathway 144.

#### Synthetic compounds

A recent study demonstrated that HDACs and the PI3K/Akt/mTOR signaling pathway were highly activated in EC cell lines and ESCC patients. As an innovative artificial small-molecule inhibitor that can suppress both the PI3K/Akt/mTOR signaling pathway and HDACs, **CUDC-907** has been reported to activate autophagy in ESCC cells by suppressing the PI3K/Akt/mTOR signaling pathway or LCN2 to induce accumulation of ROS, suggesting a potential targeted therapy option for patients with EC 145. Moreover, it has been reported that the dual PI3K/mTOR inhibitor **BEZ235** enhances antitumor activities by promoting autophagy in ESCC cells through the inhibition of the PI3K/Akt/mTOR signaling pathway 146. In addition, **BJ-B11**, a selective Hsp90 inhibitor, exhibited effective and potent antitumor activity in ESCC cells by inducing autophagy by suppressing the Akt/mTOR/p70S6K signaling pathway in a time- and concentration-dependent manner 147. Additionally, a recent study showed that the CDK4/6 inhibitor **palbociclib** can effectively improve the radiosensitivity of ESCC cells *in vivo* and *in vitro* by suppressing mTOR and thus has great potential to act as a radiosensitization agent for ESCC treatment 148 **(Table 3)**.

#### Natural compounds

An active component of licorice named **echinatin** was found to promote autophagy in ESCC cells through suppressing the Akt/mTOR signaling pathway, resulting in sensitization of ESCC cells to 5-FU treatment 149. In addition, as a bioactive component derived from Panax notoginseng and ginseng, it was reported that **ginsenoside Rk3** could exhibit a significant inhibitory effect on the proliferation and colony formation of ESCC cells by activating autophagy through the inhibition of the PI3K/Akt/mTOR signaling pathway. Experiments with the KYSE150 xenograft model demonstrated that **Rk3** significantly suppressed tumor growth and caused little organ toxicity, suggesting that **Rk3** may be a promising antitumor agent for EC 150. Furthermore, **ursolic acid (UA)** has demonstrated effectiveness in inhibiting the growth and metastasis of ESCC cells by inducing autophagy mediated by reactive oxygen species (ROS) through the inhibition of the PI3K/Akt/mTOR signaling pathway 151. **Dihydroartemisinin (DHA)**, the main active derivative of artemisinin, has been documented to induce autophagy in a dose-dependent manner by reducing Akt phosphorylation and suppressing the Akt/mTOR signaling pathway, which could lead to the inhibition of ESCC cell migration 152. Furthermore, the combination of **curcumin (CUR)** and **docetaxel (DTX)** was found to induce autophagy in ESCC cells through the PI3K/AKT/mTOR signaling pathway, enhancing the antitumor effect of DTX *in vivo* 153 **(Table 3).**

As the PI3K/AKT/mTOR pathway is hyperactive in EC, there are opportunities for the discovery and research of anti-EC drugs. Targeting the PI3K/AKT/mTOR pathway selectively with small-molecule compounds, such as mTOR inhibitors, PI3K inhibitors, AKT inhibitors, and dual PI3K/mTOR inhibitors, to induce autophagy in EC cells represents a promising therapeutic approach for individuals diagnosed with EC.

### Targeting ULK1

ULKs are serine/threonine protein kinases that have the ability to form complexes with multiple regulators. Among ULKs, ULK1 is a component of the ULK1 complex (ULK1-ATG13-FIP200-ATG101), which has important functions in autophagy induction. Interestingly, upregulation of ULK1 has been observed in various cancers, including non-small cell lung cancer, colorectal cancer, nasopharyngeal carcinoma, and clear cell renal carcinoma. This upregulation has been associated with treatment resistance and unfavorable prognosis 154-159. As such, inhibiting ULK1 to regulate autophagy may be an effective therapeutic strategy. A growing number of ULK1 inhibitors have been researched and reported to suppress tumor growth and metastasis and promote autophagy in various cancer types, such as **3s** 158, **SBI-0206965** 160, **WP1130** 161, **ULK-100** 162, **ULK-101** 162, **MRT67307**^163^, **MRT68921** 163, **compound 3g** 164, and **compound 6** 165. For EC, a study found that ESCC samples had higher levels of ULK1 protein than normal EC cells and tissues, as well as higher ULK1 protein stabilization. Further study showed that the overall survival time was shorter among patients with higher ULK1 expression. Using specific small interfering RNA to inhibit ULK1 expression in ESCC cell lines can block cell proliferation, which supports the idea that blocking ULK1 may be a beneficial therapy for patients with EC 166. However, there are few relevant studies assessing the effect of ULK1 inhibitors as EC therapy. Therefore, trying to regulate autophagy using existing ULK1 inhibitors that have been applied to other kinds of cancers or developing new ULK1 inhibitors is a promising direction for EC therapeutic research and may have far-reaching ramifications in the future.

### Targeting ATG proteins

Membrane PI3P produced by VPS34 recruits PI3P-binding ATG proteins and other factors contributing to the elongation of the phagophore. ATGs such as ATG16L1 play important roles in ubiquitin-like conjugation system 1 and ubiquitin-like conjugation system 2, contributing to the formation of autophagosomes. Thus, targeting ATG proteins may be useful for efficient EC treatment because it would regulate autophagy 167. As an innate immune receptor for bacteriogenic components activated by muramyl dipeptide (MDP), nucleotide binding oligomerization domain containing 2 (NOD2) was found to be decreased in EC cells, and further study demonstrated that NOD2 overexpression promoted autophagy in and inhibited the proliferation of EAC cells by acting on the ATG16L1 pathway 168. In addition, there has been very little research on the usage of ATG modulators in EC. Notably, a growing number of ATG modulators have been discovered and tested in other diseases, including various cancers, such as **tioconazole** 169, **S130** 170, **STK683963** 171 and **flubendazole** 172. Collectively, trying to regulate autophagy using existing ATG modulators or developing new ATG modulators is a novel and promising direction for EC therapeutic research and may have far-reaching ramifications in the future** (Table 3)**.

### Targeting lysosomes

Lysosomes have key functions in the growth and metabolism of cells. During degradation of the lysosomal content, the catalytic functions of lysosomal hydrolytic enzymes can only work when the pH is in the range of 4.5 to 5. At present, there are only two autophagy inhibitors approved for clinical use: **chloroquine (CQ)** and **hydroxyquinoline (HCQ)**, which can inhibit autophagy by deacidifying the lysosome and blocking autophagosome‐lysosome fusion. Nevertheless, the clinical utility of these drugs is restricted due to high doses of **CQ** and **HCQ** are needed for effective autophagy inhibition *in vitro,* which is difficult to accomplish in humans; in addition, there is a lack of selectivity, and there are side effects 173. Notably, some new drugs targeting lysosomes have been discovered, such as **Lys05** 174,175, **mefloquine** 176,177,** VATG-027** 178, **DQ661** 179, **bafilomycin A1** 180,181, and **Ganoderma lucidum polysaccharide (GLP)** 182. For EC, **Obatoclax**, a newly developed drug with potential as an innovative anticancer agent, was observed to become sequestered within lysosomes. This resulted in the accumulation of LC3 II and p62 proteins, indicating disruption of the autophagosome-lysosome fusion process. The resulting IC_50_ values were 0.13 and 0.24 µM in HKESC-1 and EC109 cells (ESCC cancer cell lines), respectively 173,183. Moreover, a new autophagy inhibitor extracted from Panax ginseng named **ginsenoside Ro (Ro)** can effectively inhibit the fusion of autophagosomes and lysosomes by upregulating the pH in lysosomes and decreasing lysosomal cathepsin activity. This mechanism suppresses autophagy in a variety of EC cell lines, indicating that **Ro** might serve as an anticancer agent to overcome chemoresistance because of its role as an autophagy inhibitor 184. In addition, **N-(cyclohexylmethyl)-5-(((cyclohexylethyl)amino)methyl)-2-((4-(trifluoromethyl)benzyl)oxy)benzamide (4 d)**, a synthesized compound, was found to inhibit autophagy by suppressing the autophagosome-lysosome fusion process, which enhanced the chemosensitivity of vincristine in vincristine-resistant ESCC cells, thus serving as a novel autophagy inhibitor^185^. Thus, using existing lysosome inhibitors that have been applied in other kinds of cancers or developing new lysosome inhibitors to improve the outcomes of patients with EC may be promising options **(Table 3)**.

### Targeting other autophagy regulators

EC treatments directed at new targets related to autophagy have also been a focus of research in recent years in addition to the above targets. Lectin-like oxidized low-density lipoprotein receptor-1 (LOX-1) has been reported to be overexpressed in EC tissues. A sulfated polysaccharide named **fucoidan** has been reported to activate autophagy in ESCC cells by inhibiting LOX-1^186^. Both *in vitro* and *in vivo* studies have demonstrated that radiation-induced autophagy plays a protective role in preventing cell death. When ESCC EC9706 cells were treated with 10 mM **3-MA** and ionizing radiation, the sensitization enhancement ratio was 1.76, and the effect was proven in a xenograft ESCC mouse model *in vivo,* in which there was an obvious decrease in tumor volume relative to that achieved by single treatment. Unfortunately, the poor solubility profile of **3-MA** necessitates administration of high doses to obtain sufficient autophagy inhibition 187,188 Targeting these autophagy-related targets and pathways to regulate autophagy in EC cells may help develop novel therapeutic strategies for patients with EC **(Table 3)**.

### LncRNAs regulating autophagy in EC

A study identified downregulation of miR-149-5p and upregulation of lncRNA NFIB and DRAIC in EC cells. Subsequent investigations have revealed that targeting the miR-149-5p/NFIB axis by inhibiting DRAIC can induce autophagy in EC cells, leading to suppressed proliferation and invasion of ESCC cells. Consequently, DRAIC emerges as a potential key gene for diagnosis and treatment of EC 189. Furthermore, lncRNAs are involved in the regulation of drug resistance in EC. A report has shown that **lncRNA LINC00337** is overexpressed in ESCC cells and tissues. Downregulating **LINC00337** inhibited autophagy in ESCC cells and enhanced sensitivity to **cisplatin** via the upregulation of TPX2 by recruiting E2F4^190^. In the context of EC treatment, regulating autophagy in EC cells by targeting lncRNAs has been shown to be a novel and promising approach **(Table 6)**.

## Targeting ferroptosis pathways with small-molecule compounds in EC

### Brief introduction to ferroptosis

**Figure 4:**
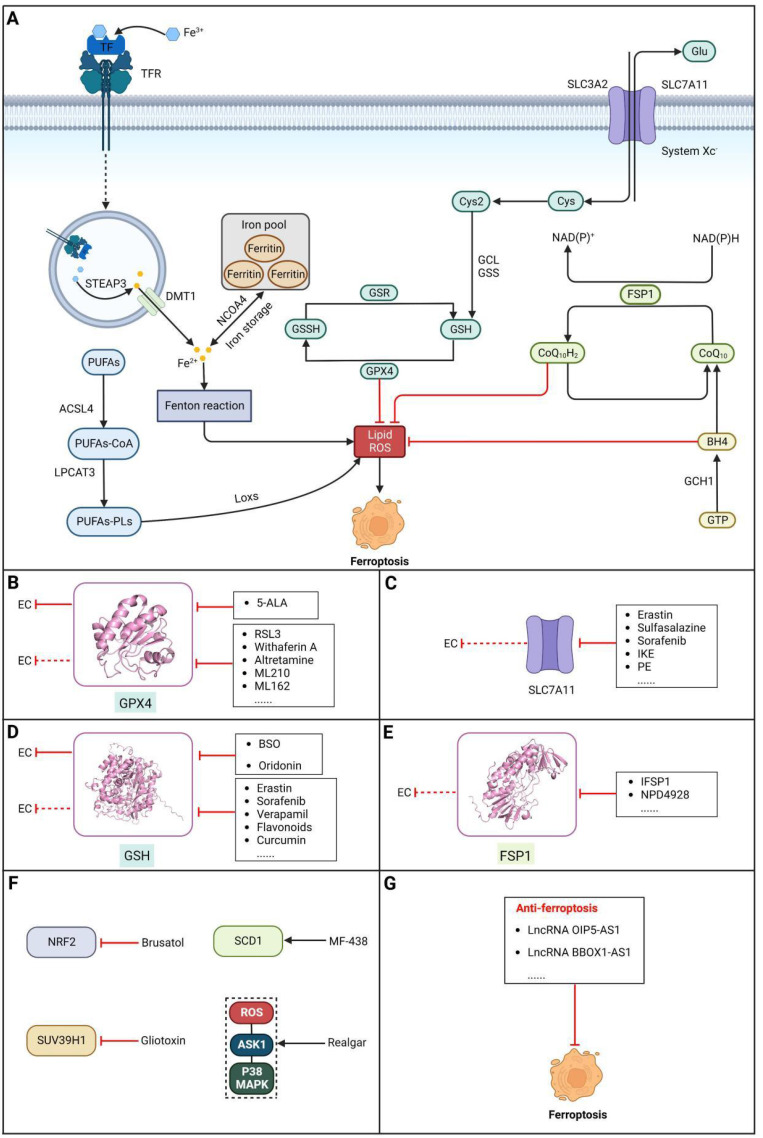
** Core ferroptosis pathways in EC and EC therapeutic approaches by targeting ferroptosis pathways.** (A) Core ferroptosis pathways in EC. Ferroptosis is an iron- and lipid ROS-dependent form of RCD, which is determined by several pathways, including pathways related to iron metabolism, the system Xc-GSH-GPX4 pathway, pathways related to lipid peroxidation, the FSP1-NADPH-CoQ10 pathway and the GCH1-BH4 pathway. (B) Small-molecule compounds targeting ferroptosis-related GPX4 in EC and other cancers. (C) Small-molecule compounds targeting ferroptosis-related SLC7A11 in other cancers. (D) Small-molecule compounds targeting ferroptosis-related GSH in EC and other cancers. (E) Small-molecule compounds targeting ferroptosis-related FSP1 in other cancers. (F) Small-molecule compounds targeting ferroptosis-related other regulators in EC. (G) Some lncRNAs targeting autophagy-related pathways in EC. (Created with BioRender.com)

Ferroptosis, proposed by Dixon in 2012, is an iron- and lipid ROS-dependent form of RCD with ruptured outer mitochondrial membrane, condensed mitochondrial membrane, reduced mitochondrial volume and diminished or vanished mitochondrial cristae as the main morphological features 192. Ferroptosis has been extensively associated with numerous diseases, particularly cancer. Based on accumulating evidence, several pathways are related to the regulation of ferroptosis, including pathways related to iron metabolism, the system Xc-GSH-GPX4 pathway, pathways related to lipid peroxidation, the FSP1-NADPH-CoQ10 pathway and the GCH1-BH4 pathway **(Figure 4)**. Ferroptosis is an iron- and lipid ROS-dependent type of RCD. Nevertheless, some types of cancer cells can resist ferroptosis via at least three mechanisms, including restricting the availability of labile iron, limiting the synthesis and peroxidation of PUFA-PL and upregulating cellular defense systems against ferroptosis 193-195. Thus, how to breakdown antioxidant defenses to induce ferroptosis in tumor cells has garnered great interest in cancer research communities. Recently, there have been numerous studies on designing and developing anticancer drugs that induce ferroptosis in cancer cells. In this context, it is becoming increasingly clear that inducing ferroptosis in EC cells may be a feasible and practical strategy to improve clinical therapy and give hope to patients with EC.

### Targeting GPX4

Glutathione peroxidase 4 (GPX4), an antioxidative enzyme, acts as a critical negative regulator of ferroptosis due to its ability to prevent membrane lipid peroxidation 196. According to a study by Hangauer *et al.*, persistent drug-resistant cancer cells acquire dependency on GPX4, indicating that preventing acquired drug resistance may be achievable by targeting GPX4 197. Thus, inducing ferroptosis in cancer cells via inactivation or degradation of GPX4 is being intensely pursued as a novel cancer treatment strategy. A growing number of experimental compounds that can inhibit GPX4 to induce ferroptosis in cancer cells and/or certain normal cells have been identified, such as **RSL3**, **withaferin A**, **altretamine**, **ML210**, **ML162** and some diverse pharmacological inhibitor (DPI) compounds, and these ferroptosis inducers (FINs) inhibit GPX4 activity by covalently and irreversibly binding the selenocysteine (Sec) in its active site, resulting in the buildup of lipid peroxides, ultimately culminating in ferroptosis^198^. Among these compounds, it is noteworthy that the FDA has granted approval for **altretamine** to treat ovarian cancer, which could induce ferroptosis via GPX4 inhibition 199. For EC, a recent study demonstrated that ESCC cells escape ferroptosis caused by elevated lipid peroxidation by upregulating GPX4 and SLC7A11, indicating that targeting GPX4 to block this intrinsic protective mechanism against ferroptosis has great potential for the treatment of EC 200. In a recent study, an unfavorable prognosis was correlated with increased GPX4 expression and decreased HMOX1 expression. The induction of ferroptosis in ESCC cells by **5-Aminolevulinic acid (5-ALA)** was demonstrated through the regulation of GPX4 and HMOX1, which was further validated *in vivo* 201. However, although the initial* in vitro* results are promising, the poor pharmacokinetic properties of most GPX4 inhibitors remain a major barrier to their use *in vivo* 202. Moreover, some research has confirmed that GPX4 is important for the development of mice, and inactivation of GPX4 can induce acute renal failure or even death in mice 203,204. Hence, it is imperative to develop and optimize GPX4 inhibitors with improved pharmacokinetics and selectivity for clinical use **(Table 4)**.

### Targeting SLC7A11

The cystine/glutamate transporter (also known as system Xc^-^) imports extracellular cystine into cells in exchange for intracellular glutamate (Glu) at a ratio of 1:1. Once in cells, cystine (Cys2) can be converted into cysteine (Cys), which is then used to generate glutathione (GSH) in a reaction catalyzed by glutathione synthetase (GSS) as well as glutamate-cysteine ligase (GCL) 196,205. As a core component of system Xc^-^, solute carrier family 7 member 11 (SLC7A11, also known as xCT) promotes the uptake of cystine and the biosynthesis of glutathione, thereby terminating the lipid peroxidation reaction and inhibiting the occurrence of ferroptosis. Interestingly, because tumor cells are often exposed to high levels of oxidative stress, SLC7A11 is more important for enhancing their antioxidant defense and inhibiting ferroptosis, which is beneficial for tumor growth 206. Extensive research has consistently demonstrated the overexpression of SLC7A11 in several cancer types, including colorectal cancer, ovarian cancer, lung cancer, and ESCC. This upregulation of SLC7A11 is associated with the occurrence, progression and drug resistance of tumors 207,208. Similarly, a recent study demonstrated that overexpression of SLC7A11 was linked to poorer survival and therapy outcomes among patients with ESCC 207. Therefore, SLC7A11 is regarded as a promising target in therapy for different cancers, including EC. Some compounds have been identified and validated as SLC7A11 inhibitors, including but not limited to **erastin**, **sulfasalazine**, **sorafenib**, **imidazole ketone erastin (IKE)** and **piperazine erastin (PE)** 192,196,209. For EC, as mentioned above, a recent study found that damage from increased lipid peroxidation can be avoided by upregulating GPX4 and SLC7A11, which rescues ESCC cells from ferroptosis, indicating that targeting SLC7A11 to block this intrinsic protective mechanism against ferroptosis has great potential in the treatment of EC 200. Undoubtedly, SLC7A11 is a promising target in EC therapy. However, all currently available SLC7A11 inhibitors have either bioavailability issues or off-target effects, which limit the clinical application of these inhibitors 210. To overcome these limitations, packaging and delivering these drugs with biocompatible nanoparticles might be a good strategy, as it not only increases their bioavailability and efficacy but also may reduce or eliminate associated side effects 211**.**

### Targeting GSH

Glutathione (GSH), a natural tripeptide that includes cysteine, glycine and glutamic acid, is a pivotal intracellular antioxidant that can scavenge reactive oxygen species (ROS), causing the maintenance of cellular homeostasis. In contrast to normal cells, most cancer cells tend to have an elevated level of ROS to support their survival, metastasis, proliferation and growth in different conditions or microenvironments, and this can be exploited in cancer therapy 212,213. Correspondingly, the expression of GSH is elevated in some cancers, such as lung cancer, head and neck cancers, breast cancer and ovarian cancer 214. Further studies have confirmed that GSH is not only a pivotal regulator of cancer cell metastasis, progression and development but also is involved in resistance to chemotherapeutic drugs 215,216. Hence, many researchers believe that depleting intracellular GSH might be a useful strategy to increase oxidative stress in tumor cells and improve cancer therapy outcomes. Various strategies have been proposed to reduce intracellular levels of glutathione (GSH), considering the therapeutic needs of cancer treatment and the processes involved in GSH metabolism, including 1) blocking the supply of raw materials for GSH synthesis (e.g., **erastin, imidazole ketone erastin, sorafenib and sulfasalazine)** 192,196,209; 2) delivering certain substances to consume GSH; 3) promoting GSH efflux (e.g., **verapamil**, **flavonoids** and **staurosporine**) 217-219; and 4) inhibiting GSH synthesis or regeneration (e.g., **BSO, sulfinosine** and **curcumin**) 220-222. For EC, ESCC cells were found to have high enrichment of the GSH metabolism pathway compared to normal cells, and the ESCC tumor burden in mice was greatly relieved by depleting GSH using a **γ-glutamyl cysteine synthetase inhibitor (BSO)** 223. Researchers have considered terpenoids due to their excellent pharmacological effects, including antitumor, antioxidant, and anti-inflammatory effects 224. One such compound is called **oridonin (ORI)**. The combined use of **BSO** and **ORI** was found to cause irreversible ROS accumulation and cell death by depleting GSH in ESCC cells 225. Similarly, the combined use of **ORI** and **cisplatin (CIS)** was proven to synergistically inhibit the proliferation of ESCC cells and induce cell death mediated by GSH/ROS systems 226. In addition, a recent study also suggested that **ORI** could indeed cause dysfunction of GSH synthesis, which further contributed to ferroptosis in TE1 cells 227. Based on the reports and cases discussed above, using drugs that function via different strategies to exhaust intracellular GSH has great potential in therapy for different cancers, including EC. However, long-term administration can cause increased synthesis or regeneration of GSH in cells, which may induce drug resistance 228. The problem may be solved by using strategies such as simultaneously inhibiting GSH synthesis from the upstream pathway and consuming existing GSH within cancer cells. In addition, most drugs that deplete GSH, for example, **BSO,** have short half-lives (less than 2 hours) and are usually abundantly enriched in normal tissues. Therefore, there is an urgent need for strategies to enhance the targeted delivery efficiency and bioavailability of free drugs. Nanomedicine may be a means to solve these problems and is gaining increasing attention for its use in synergistic cancer treatment and GSH depletion; for example, manganese dioxide-based nanocarriers, gold nanoclusters, iron-linked nanoscale metal-organic frameworks and polyoxometalate-based nanoplatforms have been tested and have shown a good ability to simultaneously deliver therapeutic components and GSH-depleting agents 229. In summary, improved strategies targeting GSH with small-molecule compounds may be promising for patients with EC **(Table 4)**.

### Targeting FSP1

Ferroptosis suppressor protein 1 (FSP1) acts as an oxidoreductase by reducing CoQ10 to CoQ10H2, which can function as a lipophilic radical trap within membranes, inhibiting ferroptosis. Thus, FSP1 is a major ferroptosis defense factor. A study has demonstrated the widespread expression of FSP1 in a majority of cancer cell lines. Moreover, the FSP1 inhibitor iFSP1 exhibited significant efficacy in sensitizing these cells to ferroptosis induced by RSL3 230. Furthermore, Yoshioka et al. screened a chemical library and identified a compound called **NPD4928** that could induce ferroptosis by directly inhibiting FSP1 and enhancing the sensitivity of a variety of cancer cells to GPX4 inhibitors 231. At present, few modulators of FSP1 have been studied and identified. In addition, few studies of how FSP1 inhibitors affect EC therapy have been conducted. However, the previously mentioned studies targeting FSP1 with small-molecule compounds have shown great potential for efficient EC treatment.

### Targeting other factors related to ferroptosis

New targets based on ferroptosis have also been a focus of EC treatment in recent years along with the abovementioned targets. A natural compound called **gliotoxin** has shown antitumor properties in a variety of cancers. In a recent study, it was revealed that gliotoxin induced ferroptosis in ESCC cells through the downregulation of SUV39H1 expression. This finding suggests the potential of gliotoxin as a novel natural therapy for ESCC 232. In addition, **realgar (REA)**, a Chinese herbal decoction, was found to not only decrease the proliferation, migration and invasion of KYSE150 and Eca109 cells but also promote ferroptosis by activating the ROS-ASK1-p38 MAPK signaling pathway 233. Moreover, high expression of NRF2 protein in both primary tissues and EAC cell lines was detected by Ballout *et al.*, and further studies showed that treatment with the NRF2 inhibitor **brusatol** alone or in combination with cisplatin induced ferroptosis of EAC cells by targeting NRF2; these results were evidenced by reduced expression of the ferroptosis markers GPX4 and xCT, which was confirmed *in vivo* 234. Furthermore, analyses revealed that stearoyl-CoA desaturase (SCD1) conferred radiation resistance by inhibiting ferroptosis in ESCC cells and was related to unfavorable survival in patients with ESCC. The utilization of **MF-438**, an inhibitor of SCD1, for targeting SCD1 has been demonstrated to substantially enhance the radiosensitivity of ESCC cells through the induction of ferroptosis 235. These findings provide additional potential novel therapeutic ferroptosis-related targets and pathways for the treatment of EC and may have far-reaching ramifications in the future **(Table 4)**.

### LncRNAs regulating ferroptosis in EC

One recent study found that the expression of both **lncRNA OIP5-AS1** and its direct target GPX4 was upregulated in ESCC cells, and subsequent experiments confirmed that knockdown of lncRNA OIP5-AS1 promoted ferroptosis in ESCC cells by regulating GPX4^236^. Additionally, there have been reports indicating a significant upregulation of **long non-coding RNA BBOX1 antisense 1 (BBOX1-AS1)** in ESCC tissues, with this upregulation being associated with an unfavorable prognosis. Further study proved that downregulation of BBOX1-AS1 suppressed cell proliferation and metastasis and promoted ferroptosis in ESCC cells by upregulating miR-513a-3p to inhibit the expression of SLC7A11 237. The lncRNAs involved in ferroptosis in EC cells deserve further study, which may uncover potential therapeutic targets for the treatment of patients with EC **(Table 6)**.

## Targeting pyroptosis pathways with small-molecule compounds in EC

### Brief introduction to pyroptosis

**Figure 5:**
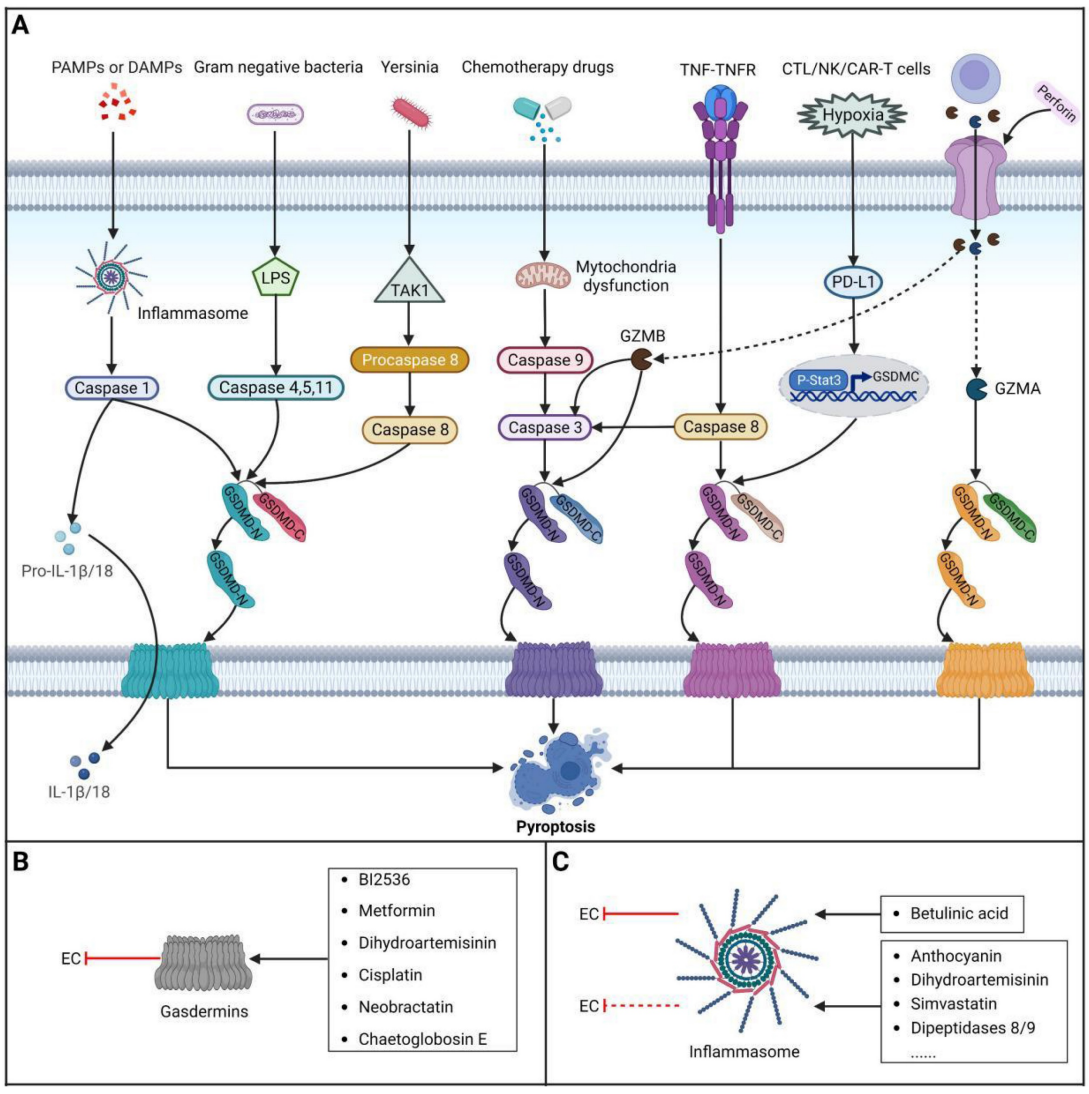
** Core pyroptosis pathways in EC and EC therapeutic approaches by targeting pyroptosis pathways.** (A) Core pyroptosis pathways in EC. Pyroptosis is a lytic and inflammatory type of RCD caused by intracellular or extracellular factors, such as toxins, viruses, bacteria and chemotherapy drugs, which is determined by several pathways, including the canonical pathway, the noncanonical pathway, the granzyme-mediated pathway and the caspase-3/8-mediated pathway. (B) Small-molecule compounds targeting pyroptosis-related gasdermins in EC. (C) Small-molecule compounds targeting pyroptosis-related inflammasomes in EC and other cancers. (Created with BioRender.com)

Pyroptosis is a lytic and inflammatory type of RCD usually caused by intracellular or extracellular factors including chemotherapy drugs, toxins, viruses and bacteria, leading to cell swelling, chromatin fragmentation, plasma membrane lysis and secretion of proinflammatory cytokines such as IL-18 and IL-1β. First observed in 1992 in infected macrophages by Zychlinsky et al., pyroptosis was subsequently identified as a gasdermin (GSDM)-mediated type of PCD in 2015 238,239. To date, four different pathways have been identified to be involved in pyroptosis: the caspase-3/8-mediated pathway, the granzyme-mediated pathway, the canonical pathway and the noncanonical pathway 240 **(Figure 5).** Pyroptosis is considered to play roles in both tumor progression and tumor suppression. On the one hand, intense, acute activation of pyroptosis causes massive infiltration of immune cells, which represses tumor development by directly inducing substantial cancer cell death and activating antitumor immunity. On the other hand, long-term chronic pyroptosis of cancer cells induced by an unfavorable TME is more conducive to tumor progression 241. Therefore, pyroptosis-based therapies may be promising cancer therapies because they induce intense, acute pyroptosis in cancer cells. To date, several methods for improving therapeutic efficacy by targeting pyroptosis or combining pyroptosis-targeted agents with other cancer treatment methods have been explored. In this context, it is becoming increasingly clear that targeting pyroptosis with small-molecule compounds has great potential to improve therapeutic outcomes for patients with EC.

### Targeting gasdermins

The GSDM family is composed of six members in humans: gasdermin A (GSDMA), gasdermin B (GSDMB), gasdermin C (GSDMC), gasdermin D (GSDMD), gasdermin E (GSDME) and DFNB59. Except for DFNB59, all members are made up of two conserved domains: the GSDM-CT (C-terminal) and GSDM-NT (N-terminal) domains. The activity of GSDM-NT can be inhibited by GSDM-CT via FLTD peptides (linkers). Once the connection is cleaved by an activation signal, plasma membrane pores are formed by GSDM-NT, which causes pyroptosis. As a family of pore-forming proteins, GSDMs are regarded as effectors of pyroptosis, and activation of GSDMs may improve the outcomes of cancer therapy. Among all GSDMs, GSDMD and GSDME are two effectors that have been extensively studied in pyroptosis. Previous studies have shown that some drugs or molecules can trigger GSDM-mediated pyroptosis in various cancer types, including but not limited to ovarian cancer 242, breast cancer 243, colon cancer 244, lung cancer 245,246 and melanoma 247. In line with these studies, pyroptosis can also be induced by certain drugs or molecules in ESCC cells by targeting certain GSDMs. A recent study indicated that the PLK1 inhibitor **BI2536** has the potential to trigger pyroptosis in ESCC cells by enhancing the expression of GSDME in the cytoplasm and activating the caspase-3/GSDME pathway. This leads to a noteworthy enhancement in the effectiveness of cisplatin. Interestingly, ESCC tissues exhibited elevated levels of GSDME expression compared to the surrounding normal tissues. Additionally, the group with higher GSDME expression demonstrated a more favorable five-year survival rate than the group with lower GSDME expression. These findings suggest that GSDME could serve as a potential prognostic factor 248. Furthermore, a report demonstrated that **metformin** could induce pyroptosis in ESCC cells by regulating the miR-497/PELP1 axis, ultimately resulting in GSDMD-mediated pyroptosis, indicating that metformin may be a potential treatment for patients with cancers resistant to chemotherapy and radiotherapy but sensitive to pyroptosis 249. In addition, GSDME was reported to be overexpressed in EC tissue. As an effective and approved treatment option for a variety of cancers, photodynamic therapy was found to promote pyroptosis in human ESCC cells by targeting the PKM2/caspase-8/caspase-3/GSDME axis 250. Moreover, a recent study showed that **dihydroartemisinin (DHA)** could promote pyroptosis in ESCC cells through the PKM2/caspase-8/caspase-3/GSDME axis, providing new insights into the role of DHA in ESCC 251. Moreover, treatment with **cisplatin** was proven to cause the activation of CAPN1 and CAPN2 upstream of BAX/BAK cleavage, leading to activation of caspase-9/3, which in turn resulted in GSDME-dependent pyroptosis in ESCC cells 252. In addition, a caged prenylxanthone named **neobractatin (NBT)** has been reported to activate GSDME-mediated pyroptosis in ESCC cells 253. Furthermore, a recent study showed that **chaetoglobosin E,** which is derived from fungal secondary metabolites, could induce GSDME-mediated pyroptosis in ESCC cells by inhibiting PLK1 and thus has the potential to improve the outcomes of EC treatment 254. In summary, these findings suggest that drugs or molecules that target GSDM-related pathways can be utilized as therapeutic options for patients with EC. Furthermore, nanomaterials are being increasingly studies as a means to better target GSDMs, as they have great potential in delivering GSDM cytotoxic peptides/expression constructs into tumors and inducing targeted activation of intrinsic GSDM pro-cell death functions in tumor cells. This may have far-reaching ramifications for treating patients with EC in the future **(Table 5)**.

### Targeting inflammasomes

Inflammasomes are multimeric cytosolic protein complexes composed of the adaptor protein apoptosis-associated speck-like protein (ASC), cytosolic pattern recognition receptors (PRRs) and pro-caspase-1 that assemble in response to danger signals, triggering caspase-1 activation and pyroptosis. In recent years, it has been proposed that improving inflammasome activity may prevent the development of related tumors. A protective role of inflammasomes has mainly been found in colitis-related cancers. NLRP3-/- mice were found to be more susceptible to acute and recurrent colitis-associated cancer 255. Furthermore, the proliferation and invasion of renal carcinoma can be inhibited by increasing AIM2 expression 256. Excitingly, some PRR activators have been reported thus far, including some that either activate PRRs directly or activate inflammasome components or related signaling events indirectly. For instance, **anthocyanin** was reported to reduce the viability of oral squamous cell carcinoma cells and inhibit migration and invasion abilities by activating NLRP3 inflammasome-based pyroptosis 257. A recent study indicated that **dihydroartemisinin** could promote pyroptosis in breast cancer cells by targeting the AIM2/caspase-3/DFNA5 pathway 258. Furthermore, **simvastatin** was proven to inhibit non-small cell lung cancer cell proliferation and migration through pyroptosis by activating the NLRP3-caspase-1 axis 259. In addition, **dipeptidases 8/9 (DPP8/9)** were reported to activate NLRP1**,** leading to pyroptosis in human acute myeloid leukemia (AML) 260. Conversely, some other reports have indicated that inflammasomes can also promote development of tumor. A report has shown that inflammasome/IL-1 pathways promote tumor cell proliferation and migration in both human and animal breast cancer models, and tumor growth and lung metastasis are significantly inhibited in mice lacking inflammasome components 261. As a result, some potential inhibitors of inflammasomes have been developed to treat cancer. Considering the aforementioned findings, how inflammasomes function in human cancer remains controversial. Inflammasome's dual effects in cancer seem to be dependent on several factors, such as expression, the stage of tumorigenesis, cancer type, and the presence of mutations affecting PRR expression and downstream effector molecules (e.g., IL-1β/18). For EC, a lupine pentacyclic triterpene compound named **betulinic acid (BA)** was found to induce pyroptosis in ESCC cells by upregulating the protein expression of ASC and caspase-1, resulting in improved sensitivity of ESCC cells to cisplatin, which was further confirmed *in vivo* 262. Because this area of study is fairly new in EC research, further studies are needed. However, the aforementioned studies utilizing small-molecule compounds to target inflammasomes have exhibited significant promise for effective treatment of EC **(Table 5)**.

## Targeting necroptosis pathways with small-molecule compounds in EC

### Brief introduction to necroptosis

**Figure 6:**
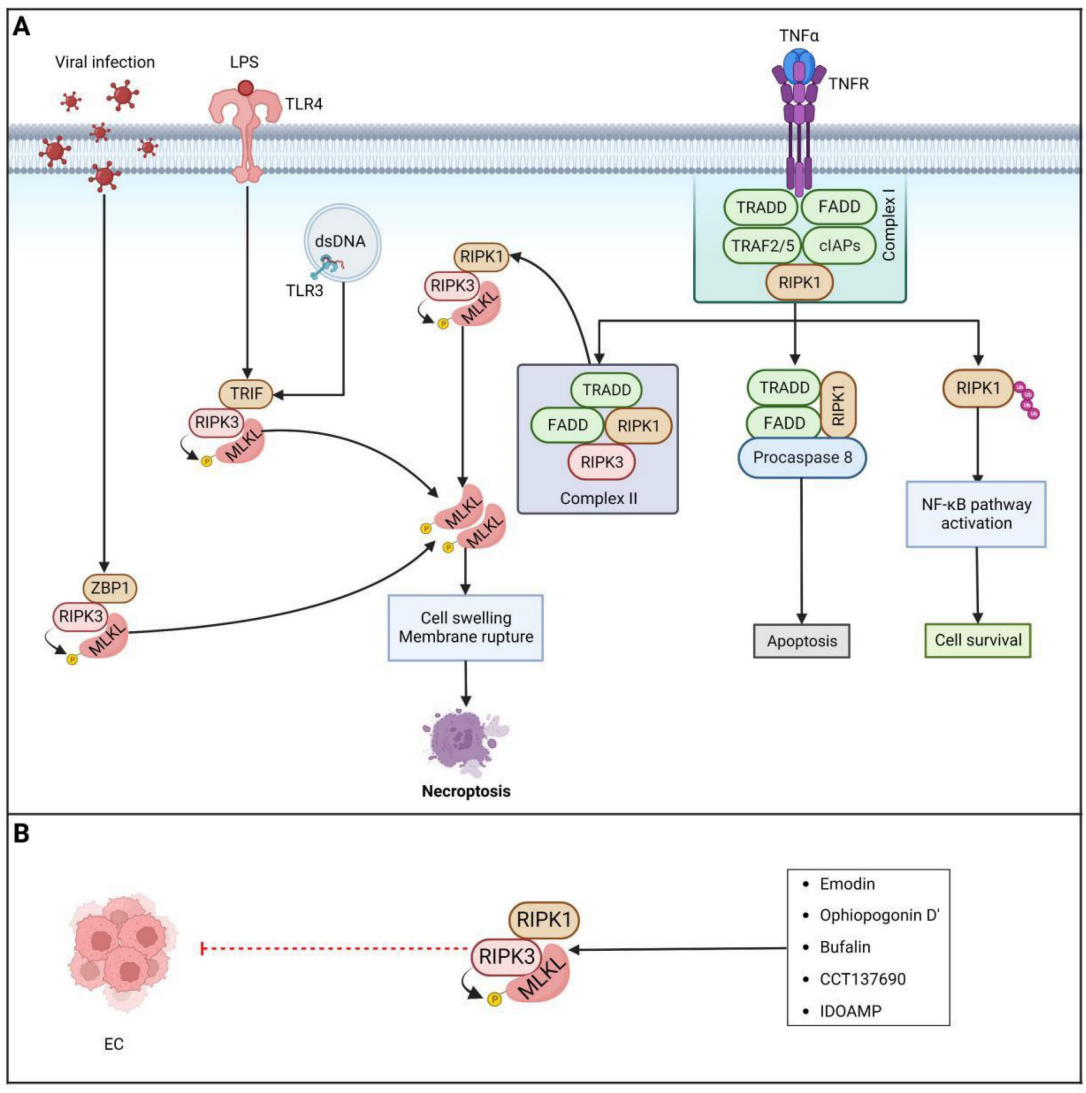
** Core necroptosis pathways in EC and EC therapeutic approaches by targeting necroptosis pathways.** (A) Core necroptosis pathways in EC. Necroptosis is a highly regulated caspase-independent form of RCD. And it is widely believed that mixed lineage kinase domain-like protein (MLKL), receptor-interacting protein kinase 1 (RIPK1) and receptor-interacting protein kinase 3 (RIPK3) are critical regulators of necroptosis. (B) Small-molecule compounds targeting necroptosis-related the RIPK1/RIPK3/MLKL axis in other cancers. (Created with BioRender.com)

Necroptosis is a highly regulated caspase-independent form of RCD that bears a morphological resemblance to necrosis and a mechanistic resemblance to apoptosis. At present, it is widely believed that receptor-interacting protein kinase 3 (RIPK3), receptor-interacting protein kinase 1 (RIPK1) and mixed lineage kinase domain-like protein (MLKL) are critical regulators of necroptosis 263,264 **(Figure 6).**

Currently, necroptosis is thought to exert dual effects in different types of cancer. On the one hand, necroptosis can serve as a "fail-safe" mechanism that protects against tumor development in the absence of apoptosis 264,265. On the other hand, there is some evidence that necroptosis can cause inflammation and promote immunosuppression and cancer metastasis 266-268. Thus, understanding how necroptosis functions in EC will enable exploration of the potential of targeting necroptosis with small-molecule compounds in patients with EC as a new EC therapy method.

### Targeting the RIPK1/RIPK3/MLKL axis

There is widespread recognition that RIPK1, RIPK3 and MLKL are critical therapeutic targets for necroptosis. It is worth noting that a growing body of research suggests that necroptosis serves to suppress tumors in most cases. Interestingly, a study of over 60 cancer cell lines found that RIPK3 expression was downregulated or silenced in two-thirds of the samples, and decreased RIPK3 expression resulted in significant suppression of RIP3-dependent activation of MLKL and downstream programmed necrosis during chemotherapy-induced cell death, indicating that cancer cells can avoid necroptosis and survive 269. Likewise, RIPK3 expression was proven to be significantly downregulated in primary colorectal cancer cells compared with paired normal colorectal mucosa cells 270. In the majority of AML samples, there was a notable decrease in RIP3 expression when compared to healthy samples 271. Moreover, RIPK3 was observed to be largely downregulated in EC, and decreased RIPK3 expression was related to a better response to chemotherapy and prolonged survival 272. Furthermore, both RIPK1 mRNA and protein were downregulated in HNSCC cell lines, which was thought to enhance protumorigenic properties such as cell migration 267. In addition, in resectable primary ovarian cancer 273, pancreatic adenocarcinoma 274, gastric cancer 275 and colon cancer 276, there was an association between decreased overall survival and disease-free survival and downregulated expression of MLKL. Therefore, inducing necroptosis by activating key regulators of necroptosis, including RIPK1, RIPK3 and MLKL, has emerged as a promising option for therapy for different cancers, including EC. Furthermore, an increasing number of compounds and some therapeutic agents have been reported to exhibit potential antitumor efficacy by manipulating necroptosis. The anthraquinone compound **emodin** has been proven to trigger necroptosis in glioma U251 cells and inhibit the proliferation of U251 cells by activating the TNF-α/RIP1/RIP3 axis 277. A natural compound extracted from Ophiopogon japonicus named **ophiopogonin D' (OPD')** was proven to induce significant necroptosis by upregulating RIPK1 in prostate cancer cells 278. Moreover, **bufalin** has been reported to induce necroptosis of human breast cancer cells by upregulating both RIPK1 and RIPK3 and to inhibit tumorigenesis in a mouse xenograft model engrafted with MDA-MB-231 cells 279. A previous study also showed that the Aurora kinase inhibitor **CCT137690** can trigger necrosis-like death of PDAC cells by activating MLKL, RIPK1 and RIPK3^280^. Furthermore, a rosin derivative known as **IDOAMP** was found to inhibit prostate cancer growth by activating the RIPK1/RIPK3/MLKL signaling pathway 281. To date, a series of necroptosis inducers have been identified, as mentioned above. Nevertheless, it is noteworthy that the majority of studies were conducted *in vitro*, and the clinical feasibility of necroptosis inducers and their selectivity in killing tumor cells remain to be further explored. In summary, activating the necroptosis pathway with various agents, compounds, and drugs seems to be a promising approach for bypassing apoptosis resistance in cancer treatment. However, there are very few relevant experimental studies in the context of EC, and further exploration is needed to specifically design corresponding drugs for patients with EC.

## Summary of EC clinical trials targeting RCD modalities

Although there have been recent numerous publications on novel activators and inhibitors of RCD subroutines, clinical trials assessing the impact of these modulators on RCD in the context of EC are still lacking and ongoing. Currently, clinical trials for patients with EC have primarily focused on modulators of apoptosis. However, as new and high-quality research articles on various RCD modalities including apoptosis ferroptosis, pyroptosis, necroptosis and autophagy continue to emerge, it is expected that more clinical trials will be conducted with the aim of understanding and exploring these modalities. Consequently, we anticipate that in the near future, there will be a greater utilization of these five RCD modalities to optimize anti-EC treatments (**Table 7**).

## Conclusion and perspectives

Currently, the current mainstream treatments for patients with EC include endoscopic resection, surgical resection, radiotherapy, chemotherapy and immuno. Patients with early-stage EC are treated primarily through surgery or endoscopic resection, and the therapeutic efficacy is astounding. In terminal cases, surgery is not sufficient and is often combined with preoperative or perioperative ChT/CRT. Although the treatment options for patients with advanced EC are constantly being optimized, the poor quality of life and poor five-year survival rate are still disappointing. In addition, increased resistance to chemotherapy and immunotherapy and decreased radiotherapy sensitivity of EC cells are becoming prominent, problems that urgently need to be solved. Targeted therapy has recently gained prominence in various cancer treatment modalities for its ability to identify and attack cancer cells precisely, which can be applied in combination with other treatment modalities including surgical resection, radiotherapy, chemotherapy. However, in comparison to other solid tumors, EC has lagged significantly in the field of the targeted therapy. Over the previous decade, the options and availability of targeted therapies for EC is limited, including those that target VEGF, HER2, EGFR and PD-1. In this context, developing new small-molecule drugs that target such RCD subroutines has emerged as a promising therapeutic strategy for patients with EC.

Since apoptosis was discovered in 1972, knowledge of the subroutines and molecular mechanisms of cell death has increased rapidly, enhancing our understanding of pathways associated with the occurrence, progression and treatment of tumors. These advances have further prompted researchers to study RCD in the field of cancer therapy. In this context, accumulating evidence has shown that precisely targeting specific cell death patterns is a promising strategy for treating EC. During the past few decades, many new nonapoptotic forms of RCDs have been discovered, including but not limited to necroptosis, NETotic cell death, ferroptosis, autophagy, pyroptosis, oxeiptosis, entosis, parthanatos, alkaliptosis, lysosome-dependent cell death and cuprotosis, each of which has a different mechanism. In this review, present a summary of the mechanisms underlying five well-studied RCD subroutines in relation to EC, including apoptosis, ferroptosis, pyroptosis, necroptosis, and autophagy. Additionally, we outline recent advancements in the field concerning the development of small-molecule compounds and lncRNA that target these RCD subroutines. In the treatment of patients with EC, different RCD subroutines may have varying roles and significance among different patients, which could depend on multiple factors, including but not limited to the stage and classification of EC, genetic characteristics and treatment goals. The heterogeneity of patients with EC means that a one-size-fits-all RCD subroutine may not be effective for all patients, which underscores the need for tailored treatment strategies. In addition, interestingly, despite RCD mostly serving to promote cell death in cancer, it is still a double-edged sword in some cases since some studies have proven that some forms of RCD subroutines, such as pyroptosis, necroptosis and autophagy, can also promote tumor growth when certain conditions are met. For instance, in the context of autophagy, in the early stages of tumor progression, autophagy functions as a protective mechanism, preserving cellular and genomic integrity. However, as the tumor advances to a more advanced stage, autophagy can transition into a survival and defense mechanism for cancer cells, supplying vital nutrients to withstand environmental stresses. The conflicting roles of such RCD subroutines pose challenges for targeted therapy in this context. Regulating autophagy, apoptosis, and necrosis has demonstrated efficacy as therapeutic approaches in EC cell lines and animal models. However, determining whether to activate or inhibit these RCD subroutines requires further investigation and careful consideration. Hence, current research should focus on how to selectively manipulate RCD to treat patients with EC. To achieve this goal, it is essential to investigate the causes and mechanisms of different effects of RCD in tumor progression and suppression.

The decision to use RCD-targeted therapies as standalone treatments or in combination with other treatment modalities is typically based on both preclinical and clinical trials. In line with existing preclinical reports, RCD-targeted therapies can not only be used alone to directly induce cell death in EC cells or inhibit their survival and growth in some cases, but also improve treatment outcomes of treatment modalities including chemotherapy, radiotherapy and targeted therapies in some other cases. This means that RCD-targeted therapies may have potential to be used individually or in combination with other treatment modalities. It's critical to note that the clinical trials assessing the impact of RCD-targeted therapies in the treatment of EC are still lacking and ongoing. Therefore, further evidence is needed to fully understand the optimal role and effectiveness of RCD-targeted modulators and their potential combinations with other treatment modalities in EC.

**Figure 7:**
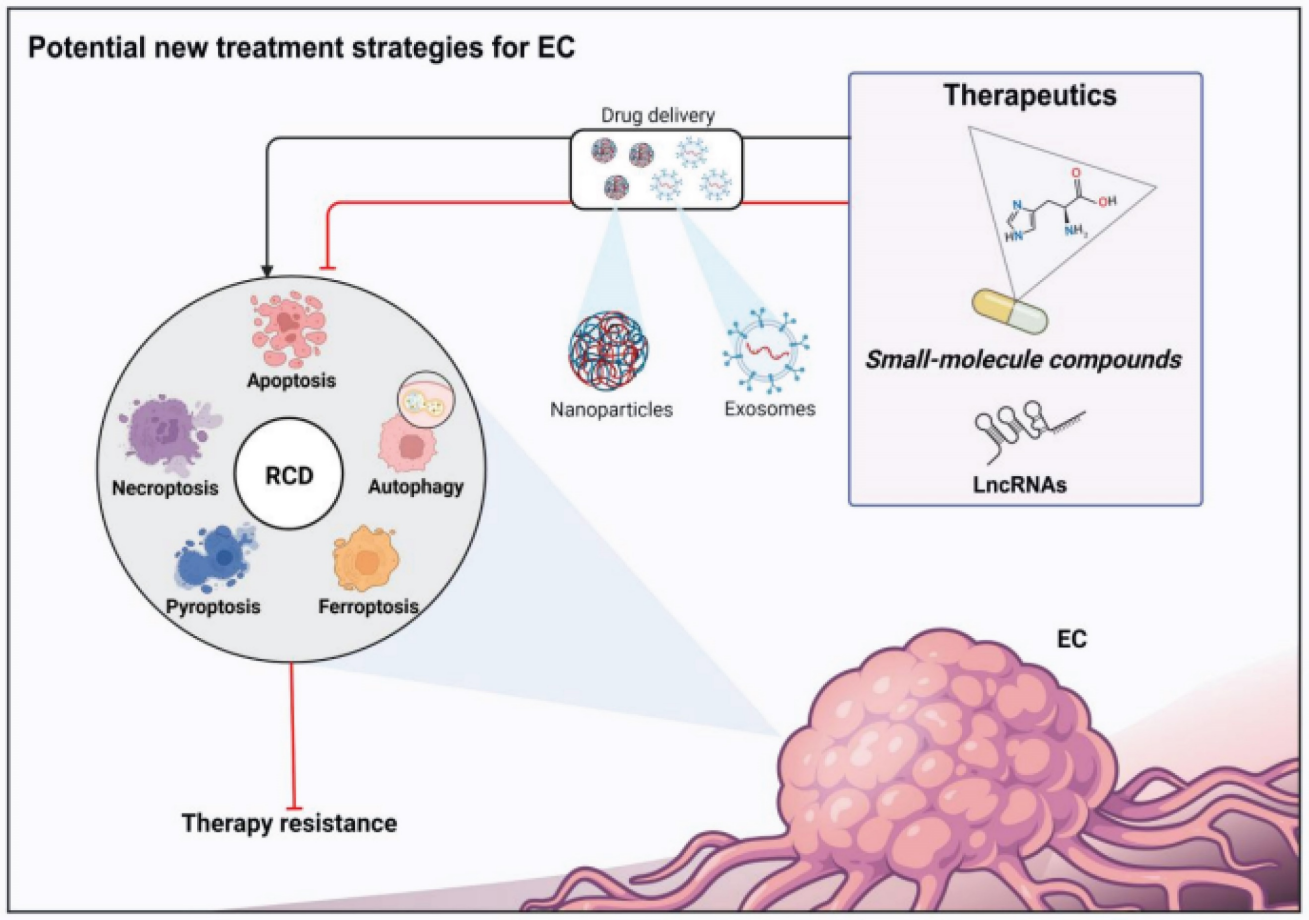
** New strategies for treating EC.** Using small-molecule compounds and lncRNAs to precisely target RCD subroutines is a promising approach to treat patients with EC, which is expected to improve the clinical outcomes of existing treatment modalities, including chemotherapy, radiotherapy and immunotherapy. And the combination of nanoparticles and exosomes is a promising future strategy for the application of small-molecule compounds and lncRNAs targeting RCD subroutines. (Created with BioRender.com)

It is crucial to understand the advantages and disadvantages of using small-molecule compounds in modulation of RCD subroutines to evaluate their potential clinical relevance and feasibility. The advantages of small-molecule compounds can easily penetrate cell membranes and target engagement due to their relatively small size, which can be designed to specifically target key molecules or pathways involved in RCD subroutines. In addition, they can not only be delivered through multiple routes, but also their pharmacokinetic properties can be optimized for efficacy and safety. However, there are still many challenges in applying these small-molecule compounds in clinical practice, especially ubiquitous bioavailability issues and off-target effects. Furthermore, the effectiveness of small-molecule compounds targeting a single target can be significantly limited by the activation of functional bypass or alternative compensatory pathways, as well as mutations in the drug target. In this scenario, dual-target or multi-target small molecules are being extensively studied as novel regimens to overcome drug resistance in cancer treatment, which have better pharmacokinetic and decreased toxic side effects and risk of drug-drug interaction. The resistance of cancer cells to a specific type of RCD may be avoided by simultaneously manipulating multiple RCD signaling pathways via dual-target or multi-target small molecules. There is a necessity to dedicate additional efforts towards the design of novel dual-target or multi-target compounds that exhibit enhanced anti-cancer activity and selectivity, aiming for more potent therapeutic effects. In addition to traditional approaches involving pharmacophore-based combination methods and structure-based modifications of existing inhibitors, the utilization of artificial intelligence (AI) in target identification and drug design has gained considerable attention. This presents a promising opportunity for enhancing the discovery of dual-target or multi-target drugs. In addition, further comprehensive investigations into the underlying mechanisms of individual RCD subroutines within cells are imperative. These studies will not only deepen our understanding of intracellular signaling molecules and the maintenance of cellular homeostasis but also facilitate the development of innovative precision-based dual-target or multi-target drugs capable of effectively eliminating EC cells. Notably, recent studies have demonstrated that nanoparticles play a crucial role in cancer therapy based on their good hydrophilicity and targeting ability and thus can be used to package and deliver the abovementioned small-molecule compounds. In addition, exosomes are also regarded as a suitable vehicle for small-molecule compounds delivery due to their low immunogenicity and high biocompatibility. To some extent, the combination of nanoparticles or exosomes is a promising future strategy for the application of small-molecule compounds and lncRNAs targeting RCD subroutines **(Figure 7).** Further research in this domain is warranted to explore its full potential in the future.

## Figures and Tables

**Table 1:** Appropriate patient population, advantages and disadvantages of different types of EC treatment

Type of EC treatment	Appropriate patient population	Advantages	Disadvantages
**Endoscopic resection**	Patients with Lesions of intraepithelial high-grade dysplasia and most T1 tumors	Minimally invasive treatment	May lead to complications (mainly perforation and bleeding).
**Surgery**	Patients with locally advanced EC (stages T1b-T4, N1-N3, M0)	Complete removal of the tumor to minimise recurrence	High surgical risk. Long postoperative recovery time. May lead to postoperative complications (such as anastomotic leakage, pulmonary complications etc.).
**Radiotherapy**	Patients with advanced EC	Can effectively control local tumors with minimal harm to healthy tissue nearby	The damage to healthy tissues may result in many side-effects (such as dysphagia, skin problems and hair loss etc.).
**Chemotherapy**	Patients with advanced EC	Can kill cancer cells anywhere in the body, even metastases	Chemoresistance. The damage to healthy tissues may result in many side-effects (such as organ damage, nausea and hair loss etc.).
**Immunotherapy**	Specific patients with advanced EC, who show favorable predictive value for immunotherapy	Has the potential for durable therapeutic effects by activating the immune system to attack EC cells	May be ineffective for certain patients. Could lead to immune-related adverse reactions.

**Table 2:** Small-molecule compounds targeting apoptosis in EC

Name	Structure	Regulatory mechanism	Function	EC subtype	Cancer cell line (activity)	References
S63845	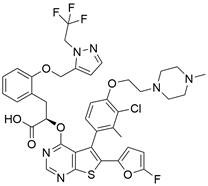	MCL-1↓, BCL-XL↓, BAX↑	Induce apoptosis	EAC	FLO-1 (IC_50_=0.8μM)	46
A1155463	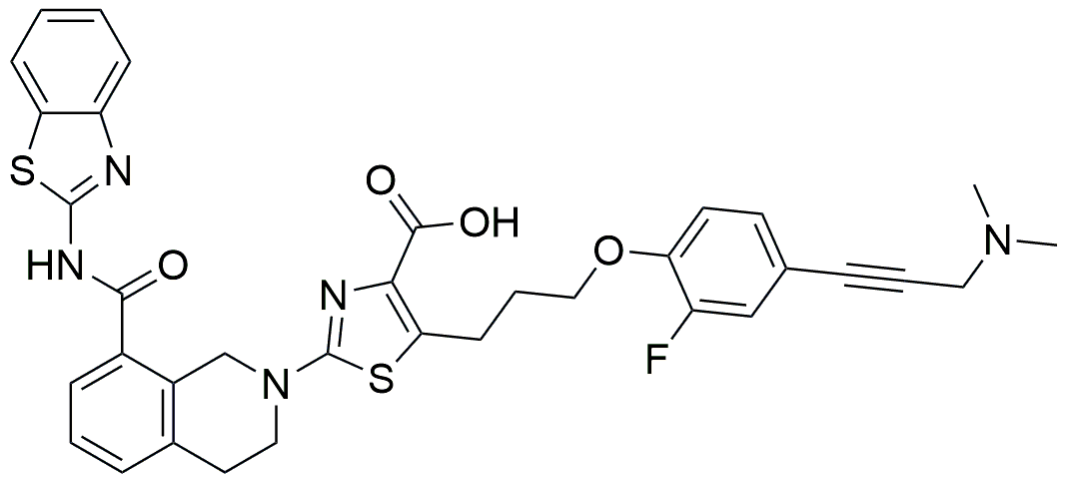	MCL-1↓, BCL-XL↓, BAX↑	Induce apoptosis	EAC	FLO-1 (IC_50_=1.13μM)	46
AT-101	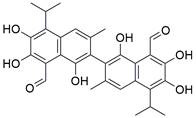	BCL-2↓, MCL-1↓	Induce apoptosis	EAC	KATOⅢ (IC_50_=3011 nM) JHESO (IC_50_=1678 nM)	47,48
ABT-263	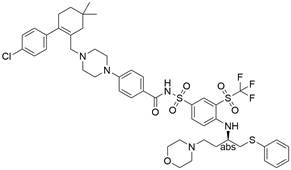	BCL-xL↓, BCL-2↓, BCL-w↓, PARP↑, caspase-3/9↑	Induce apoptosis	ESCC	EC109 (IC_50_=10.7±1.4 μM) HKESC-2 (IC_50_=7.1±1.5 μM) CaES-17 (IC_50_=8.2±1.6 μM)	49
Casticin	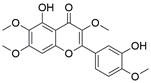	BCL-2↓, BAX↑, PARP↑, caspase-3/9↑, JNK↑	Induce apoptosis	ESCC	TE-1 ECA109	50
Pristimerin	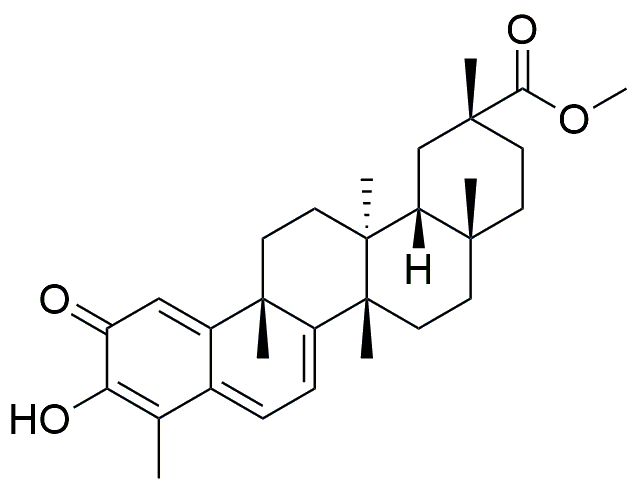	BCL-2↓, BAX↑, caspase-3/9↑	Induce apoptosis	ESCC	ECA109	51
Corilagin	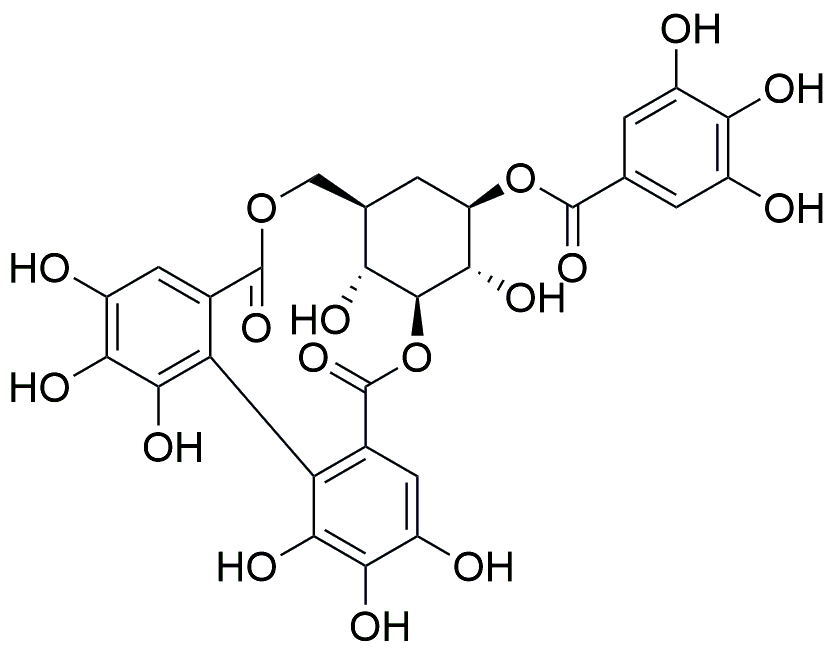	BCL-2↓, BAX↑, caspase-3/8/9↑	Induce apoptosis	ESCC	ECA109 (IC_50_=28.58 ± 2.08μM) KYSE-150 (IC_50_=35.05 ±2.86μM)	52
Matrine	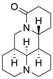	BCL-2↓, BAX↑, caspase-3/8/9↑, ROS↑	Induce apoptosis	ESCC	KYSE-150 (IC_50_=1.94 mg/ml)	53
Ch‑19	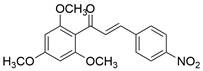	BCL-2↓, Bad↑, Bim↑, PUMA↑, BAX↑ , PARP↑, caspase-3↑, ROS↑	Induce apoptosis	ESCC	ECA109 (IC_50_=9.43μM) KYSE-450 (IC_50_=4.97μM)	54
LCL161	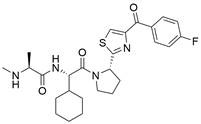	cIAP1↓, XIAP↓	Induce apoptosis	ESCC	KYSE-150 (IC_50_=34.5μM)	62,63
Tat-SmacN7	Not known	cIAP1↓, XIAP↓	Induce apoptosis	ESCC	EC109	64
CPP (+TRAIL)	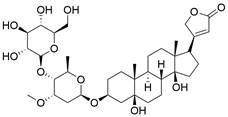	FoxP3↓, DR4↑, DR5↑	Induce apoptosis	ESCC	YES-2 KYSE-150 KYSE-510	80
Isoalantolactone	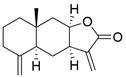	DR5↑, ROS↑	Induce apoptosis	ESCC	ECA109 EC9706 TE- 1 TE-13	81
CMSP (+TRAIL)	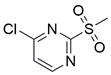	DR4↑, DR5↑, p38 MAPK↑	Induce apoptosis	ESCC	KYSE-30	82
3-DSC	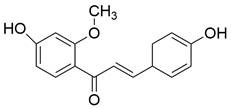	JNK/p38 MAPKs↑	Induce apoptosis	ESCC	KYSE-30 (IC_50_=19.8μM) KYSE-70 (IC_50_=12.2μM) KYSE-450 (IC_50_=24.7μM) KYSE-510 (IC_50_=24.8μM)	86
DVDMs	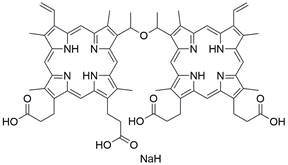	JNK/p38 MAPKs↑	Induce apoptosis	ESCC	ECA109	87
Quina	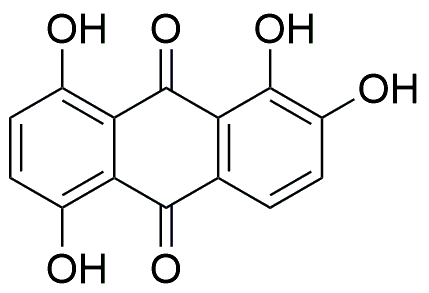	JNK/p38 MAPKs↑, STAT3↓, NF-κB↓	Induce apoptosis	ESCC	HCE-4 TE-2	88
Echinatin	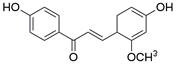	JNK/p38 MAPKs↑	Induce apoptosis	ESCC	KYSE-30 (IC_50_=15μM) KYSE-70 (IC_50_=15μM) KYSE-410 (IC_50_=6μM) KYSE-450 (IC_50_=13μM) KYSE-510 (IC_50_=10μM)	89
PPT	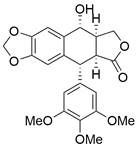	JNK/p38 MAPKs↑	Induce apoptosis	ESCC	KYSE-30 (IC_50_=0.15μM) KYSE-70 (IC_50_=0.32μM) KYSE-410 (IC_50_=0.15μM) KYSE-450, (IC_50_=0.26μM) KYSE-510 (IC_50_=0.24μM)	90
Lobaplatin	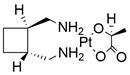	PI3K/AKT↓	Induce apoptosis	ESCC	ECA109 (IC_50_=3.99 ± 0.46μM) EC9706 (IC_50_=10.3 ± 1.9μM) KYSE-150 (IC_50_=7.91 ± 2.2μM)	92
Aprepitant	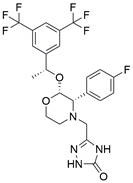	PI3K/AKT/NF-κB↓	Induce apoptosis	ESCC	KYSE-30 (IC_50_=48.21μM)	93
Ricolinostat	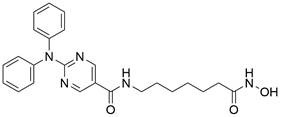	PI3K/AKT/mTOR↓, ERK↓	Induce apoptosis	ESCC	EC109 (IC_50_=46μM) KYSE-150 (IC_50_=57μM) TE-1 (IC_50_= 45 μM) TE-13 (IC_50_=37 μM)	94
VES	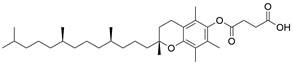	PI3K/AKT/mTOR↓	Induce apoptosis	ESCC	EC109 (IC_50_=25.1uM)	95
Psoralidin	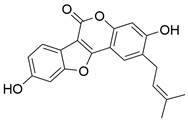	PI3K/AKT↓, NF-κB↓	Induce apoptosis	ESCC	EC9706	96
LH-20-15	Not known	PI3K/AKT/GLUT1↓	Induce apoptosis	ESCC	EC9706 (IC_50_=54.3 mg/L)	97
Oridonin	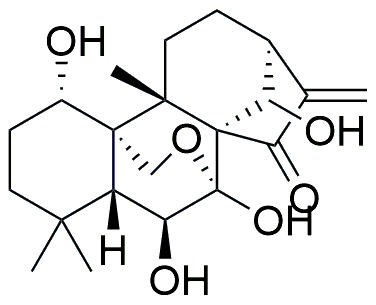	PI3K/AKT/mTOR↓, Ras/Raf↓	Induce apoptosis	ESCC	KYSE-150 (IC_50_=28.69 ± 1.45μM) EC9706 (IC_50_=34.43 ± 1.53μM) KYSE-30 (IC_50_=32.29 ± 1.51μM)	98
Cordycepin	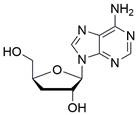	PI3K/AKT/mTOR↓, AMPK↑	Induce apoptosis	ESCC	HK (IC_50_=86.12μM) K180 (IC_50_=66.84μM) K70 (IC_50_=69.27μM) ECA109 (IC_50_=73.82μM)	99
Hinokiflavone	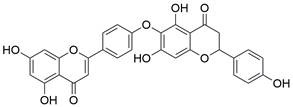	PI3K/AKT/mTOR↓	Induce apoptosis	ESCC	KYSE-150 (IC_50_=24.91μM) TE-14 (IC_50_=22.07μM)	100
Osthole	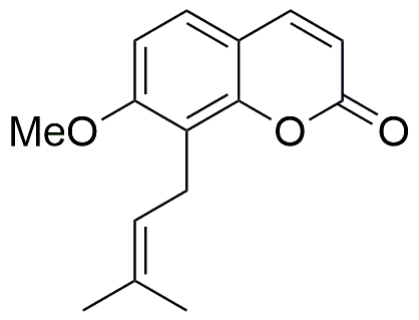	PI3K/AKT↓,	Induce apoptosis	ESCC	KYSE-150 (IC_50_=102.51μM) KYSE-410 (IC_50_=114.02μM)	101
Capilliposide C	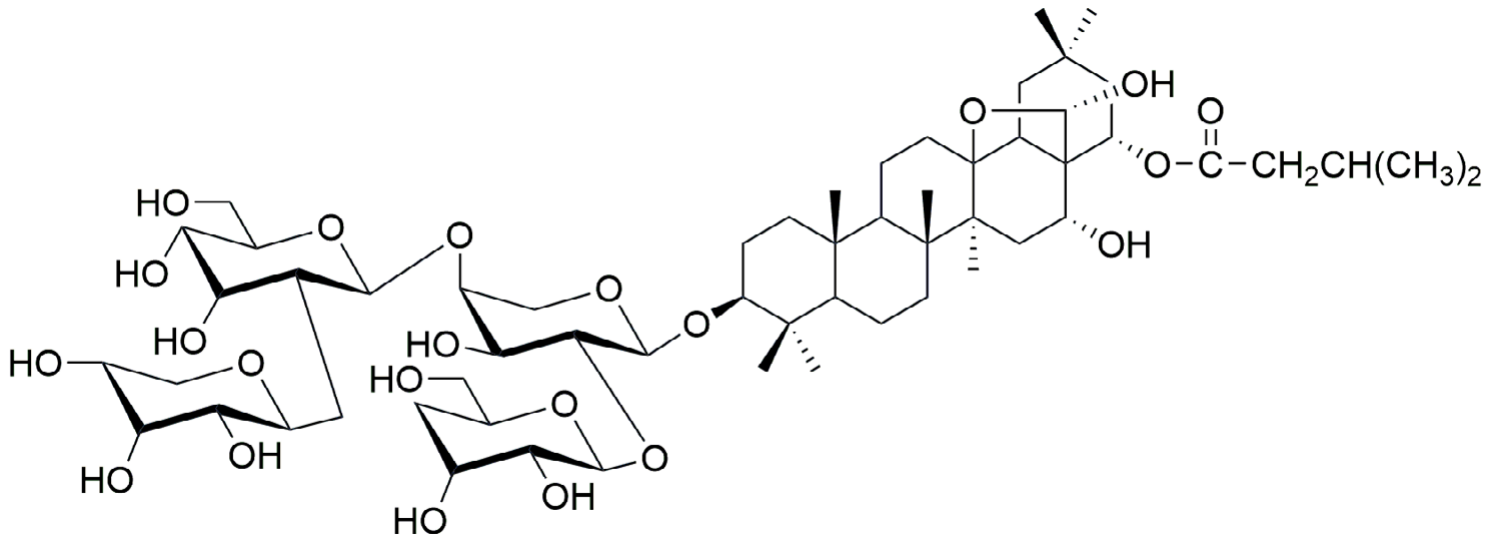	PI3K/AKT/mTOR↓	Induce apoptosis	ESCC	TE-1 (IC_50_=5.43±0.63 μM) TE-2 (IC_50_=6.64±0.91μM)	102
DEX	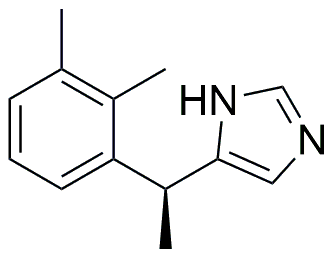	ERK↓	Induce apoptosis	ESCC	ECA109	103
APR-246	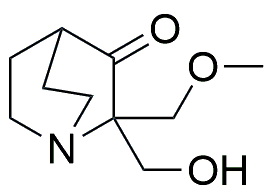	ROS↑, p73/Noxa↑	Induce apoptosis	ESCC	KYSE-410 (IC_50_=31.6μM) TE-1 (IC_50_=10.5μM)	104
Luteolin	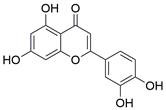	ROS/JNK↑	Induce apoptosis	ESCC	TE-1 EC109	105
Plumbagin	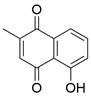	STAT3/PLK1/AKT↓	Induce apoptosis	ESCC	KYSE-150 (IC_50_=6.4μM) KYSE-450 (IC_50_=8.0μM)	106
Gossypetin	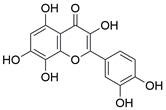	MKK3/MKK6/p38↓	Induce apoptosis	ESCC	KYSE-30 KYSE-450 KYSE-510	107
Artemisinin	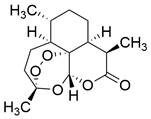	Wnt/β-catenin↓	Induce apoptosis	ESCC	EC109 (IC_50_=55.33μM)	108
Lvermectin	Not known	ROS↑, NF-κB↓	Induce apoptosis	ESCC	KYSE-70 (IC_50_=10μM) KYSE-30 (IC_50_=6μM)	109
Dehydrocostus lactone	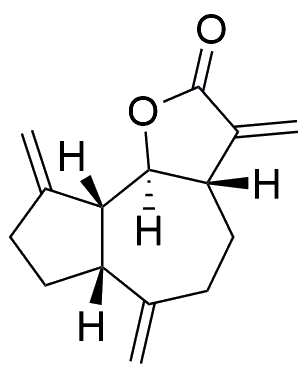	JAK2/STAT3/PLK ↓	Induce apoptosis	ESCC	KYSE-150 (IC_50_=5-10μM) ECA109 (IC_50_=5-10μM)	110
Cinobufagin	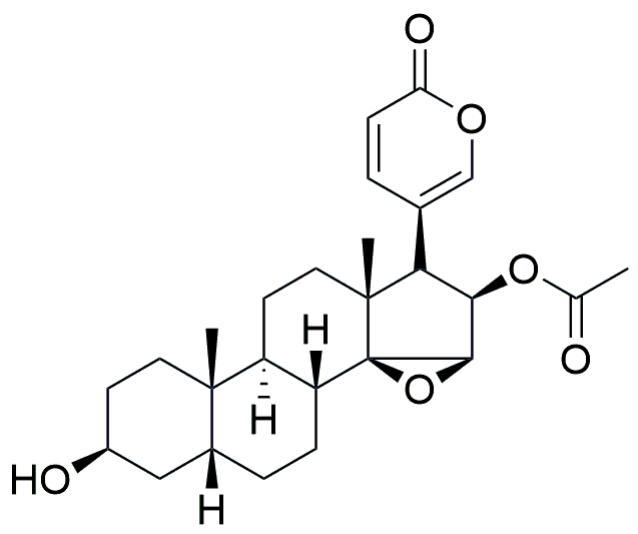	p73↑	Induce apoptosis	ESCC	EC109 (IC_50_=10.13 ± 0.02μM) KYSE-150 (IC_50_=0.09 ±0.04 μM) KYSE-520 (IC_50_=0.08 ± 0.03μM)	111
b-AP15	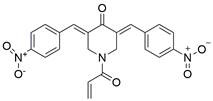	c-Myc↑, Noxa↑	Induce apoptosis	ESCC	EC1 (IC_50_=0.4-0.6μM) EC109 (IC_50_=0.1-0.2μM) KYSE-510 (IC_50_=0.1-0.2μM) KYSE-450 (IC_50_=0.4-0.6μM)	112
ACP-5862	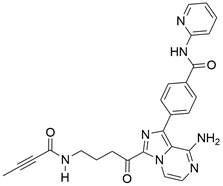	ROS/ER stress↑	Induce apoptosis	ESCC	EC109 (IC_50_=3.55μM) KYSE-270 (IC_50_=8.21μM)	113

*↓, decrease/inhibition; ↑, increase/activation

**Table 3:** Small-molecule compounds targeting autophagy in EC

Name	Structure	Regulatory mechanism	Function	EC subtype	Cancer cell line (activity)	References
CUDC-907	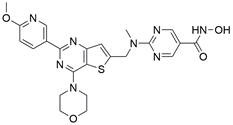	PI3K/Akt/mTOR↓, LCN2↓	Induce autophagy	ESCC	KYSE-150 (IC_50_=14.27 nM) KYSE-450 (IC_50_=6.893 nM) KYSE-510 (IC_50_=8.703 nM) KYSE-30 (IC_50_=16.95 nM)	145
BEZ235	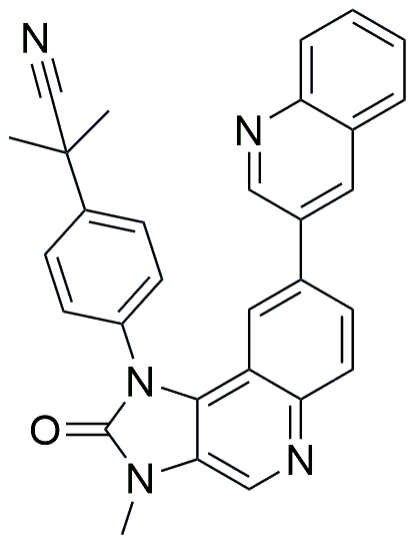	PI3K/Akt/mTOR↓	Induce autophagy	ESCC	ECA109 (IC_50_=160.7 nM) TE-1 (IC_50_=109.4 nM)	146
BJ-B11	Not known	Akt/mTOR/p70S6K↓	Induce autophagy	ESCC	ECA109 (IC_50_=0.31±0.01 μM)	147
Palbociclib	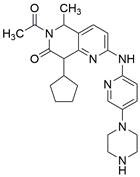	mTOR↓	Induce autophagy	ESCC	EC109 KYSE-150 KYSE-30 KYSE-70	148
Echinatin	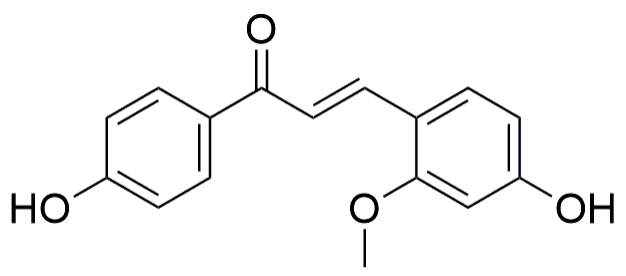	Akt/mTOR↓	Induce autophagy	ESCC	KYSE-30 KYSE-270	149
Rk3	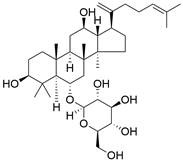	PI3K/Akt/mTOR↓	Induce autophagy	ESCC	ECA109 KYSE-150	150
Ursolic acid	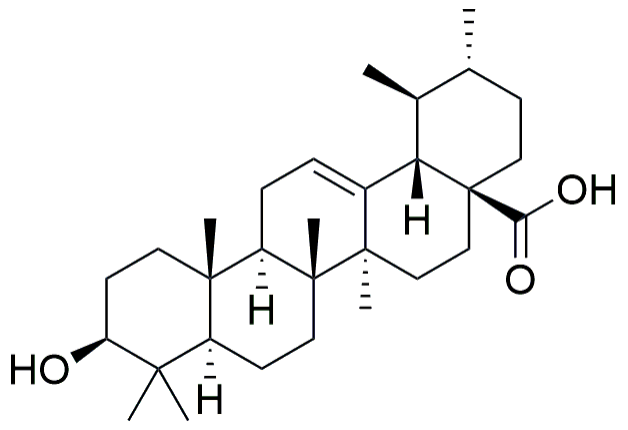	PI3K/Akt/mTOR↓	Induce autophagy	ESCC	TE-8 (IC_50_=39.01 nM) TE-12 (IC_50_=29.65 nM)	151
DHA	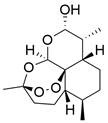	Akt/mTOR↓	Induce autophagy	ESCC	ECA109 TE-1	152
CUR(+DTX)	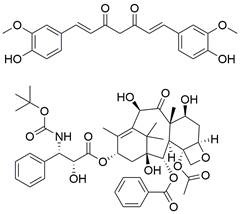	PI3K/Akt/mTOR↓	Induce autophagy	ESCC	KYSE-150 (IC_50_=5.80 ± 0.98 μg/ml) KYSE-510 (IC_50_=8.91 ± 0.88 μg/ml)	153
NOD2	Not known	ATG16L1↑	Induce autophagy	EAC	OE33 SEG-1 BIC-1	168,191
Obatoclax	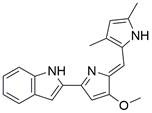	Obatoclax inhibits the fusion of autophagosomes and lysosomes	Inhibit autophagy	ESCC	EC109 (IC_50_=0.3μM)	173,183
Ginsenoside Ro	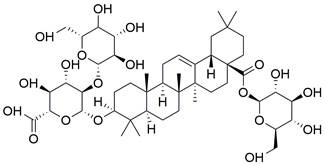	Ginsenoside Ro inhibits the fusion of autophagosomes and lysosomes	Inhibit autophagy	ESCC	EC9706 ECA109 TE-1	184
4d	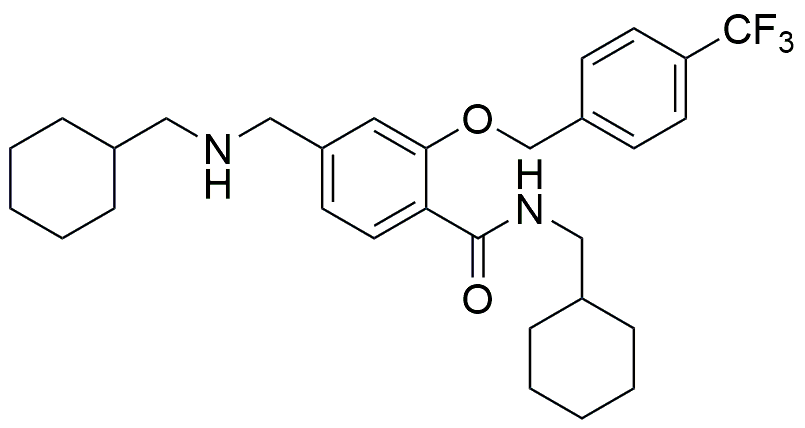	4d inhibits the fusion of autophagosomes and lysosomes	Inhibit autophagy	ESCC	ECA109	185
Fucoidan	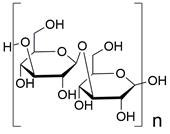	LOX-1↓	Inhibit autophagy	ESCC	TE-1 KYSE-150	186
3-MA	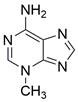	Unspecified	Inhibit autophagy	ESCC	TE-1 EC9706	187,188

*↓, decrease/inhibition; ↑, increase/activation

**Table 4:** Small-molecule compounds targeting ferroptosis in EC

Name	Structure	Regulatory mechanism	Function	EC subtype	Cancer cell line (activity)	References
5-ALA	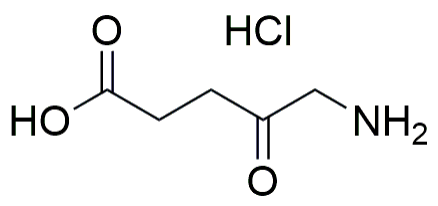	GPX4↓, HMOX1↑	Induce ferroptosis	ESCC	KYSE-30 KYSE-510	201
BSO	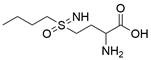	GSH↓	Induce ferroptosis	ESCC	NA	223
Oridonin	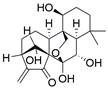	GSH↓	Induce ferroptosis	ESCC	TE-1 (IC_50_=18.95μM)	225-227
Gliotoxin	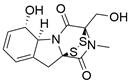	SUV39H1↓	Induce ferroptosis	ESCC	KYSE-150 TE-14	232
Realgar	Not known	ROS/ASK1/p38 MAPK↑	Induce ferroptosis	ESCC	KYSE-150 (IC_50_=48.012μM) ECA109 (IC_50_=61.336μM)	233
Brusatol	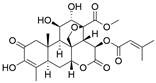	NRF2↓	Induce ferroptosis	EAC	FLO-1 (IC_50_=0.075μM) OE33 (IC_50_=0.058μM)	234
MF-438	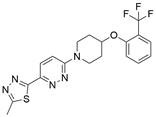	SCD1↓	Induce ferroptosis	ESCC	KYSE-30 KYSE-70 KYSE-140 KYSE-150 KYSE-410 KYSE-450 KYSE-510 (IC_50_=1-2.4μM)	235

*↓, decrease/inhibition; ↑, increase/activation

**Table 5:** Small-molecule compounds targeting pyroptosis in EC

Name	Structure	Regulatory mechanism	Function	EC subtype	Cancer cell line (activity)	References
BI2536	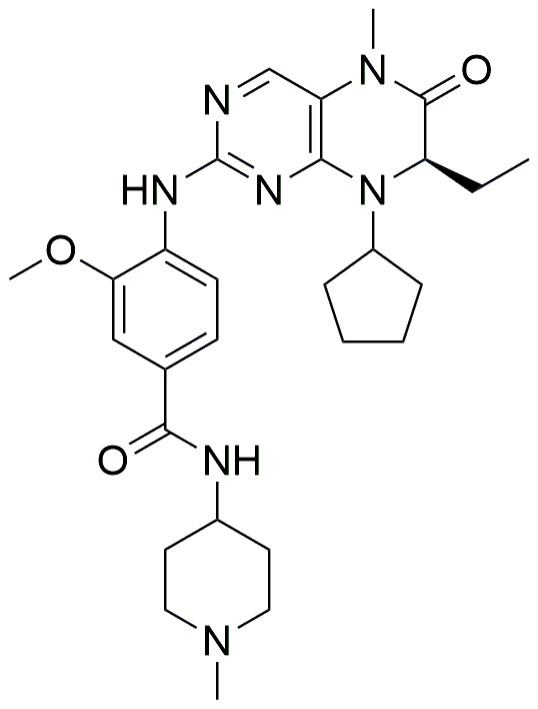	Caspase-3↑, GSDME↑	Induce pyroptosis	ESCC	YES2 KYSE-30 KYSE-70 KYSE-140 KYSE-150 KYSE-180 KYSE-410 KYSE-450 KYSE-510	248
Metformin	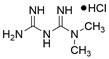	miR-497↑, PELP1↓, GSDMD↑	Induce pyroptosis	ESCC	KYSE-140 KYSE-510	249
Dihydroartemisinin	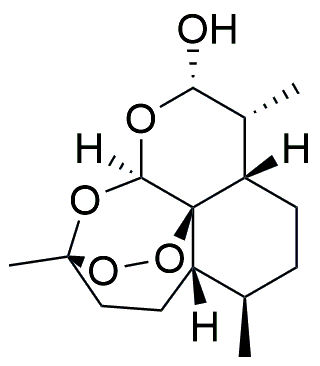	PKM2↓, Caspase-8/3↑, GSDME↑	Induce pyroptosis	ESCC	ECA109 EC9706	251
Cisplatin	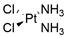	CAPN1/2↑, BAK/BAX↑, Caspase-9/3↑, GSDME↑	Induce pyroptosis	ESCC	KYSE-30 KYSE-510	252
Neobractatin	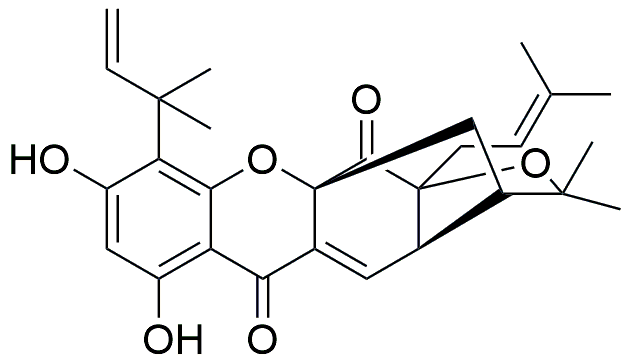	Caspase-3↑, GSDME↑	Induce pyroptosis	ESCC	KYSE-150 KYSE-450 ECA109	253
Chaetoglobosin E	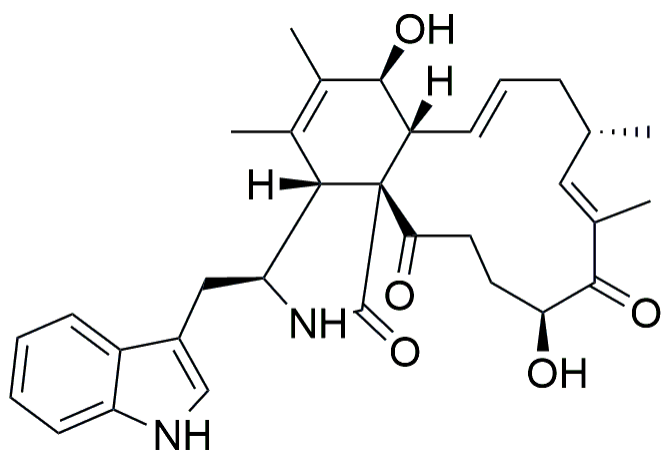	PLK1↓, GSDME↑	Induce pyroptosis	ESCC	KYSE-30 (IC_50_=2.57μM)	254
Betulinic acid	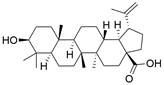	ASC↑, Caspase-1↑	Induce pyroptosis	ESCC	TE-11 (IC_50_=6-10μM)	262

*↓, decrease/inhibition; ↑, increase/activation

**Table 6:** LncRNAs targeting RCD subroutines in EC

Name	Regulatory mechanism	Function	EC subtype	References
LncRNA SNHG7	P15/16	Inhibit apoptosis	ESCC	114
LncRNA IUR	miR‑21/PTEN	Induce apoptosis	ESCC	115
LncRNA ZFAS1	miR-124/STAT3	Inhibit apoptosis	ESCC	116
LncRNA NEAT1	miR-377/E2F3	Inhibit apoptosis	ESCC	117
LncRNA FER1L4	Unspecified	Induce apoptosis	ESCC	118
LncRNA LINC01535	JAK/STAT3	Inhibit apoptosis	ESCC	119
LncRNA TUG1	miR-1294/PLK1	Inhibit apoptosis	ESCC	120
LncRNA GIHCG	miR-29b-3p/ANO1	Inhibit apoptosis	ESCC	121
LncRNA FAM83A-AS1	miR-214/CDC25B	Inhibit apoptosis	ESCC	122
LncRNA MIR22HG	STAT3/c-Myc/p-FAK	Inhibit apoptosis	EAC	123
LncRNA LINC01234	Unspecified	Inhibit apoptosis	ESCC	124
LncRNA SNHG1	miR-204/HOXC8	Inhibit apoptosis	ESCC	125
LncRNA HAGLROS	miR-206/NOTCH3	Inhibit apoptosis	ESCC	126
LncRNA TUG1	miR-498/XBP1	Inhibit apoptosis	ESCC	127
LncRNA SNHG6	miR-101-3p/EZH2	Inhibit apoptosis	ESCC	128
LncRNA CDKN2B-AS1	TFAP2A /SCN1	Inhibit apoptosis	ESCC	129
LncRNA LINC00467	miR-485-5p/DPAGT1	Inhibit apoptosis	ESCC	130
LncRNA LINC00491	Unspecified	Inhibit apoptosis	ESCC	131
LncRNA PVT1	miR-145/FSCN1	Inhibit apoptosis	ESCC	132
LncRNA GAS5	miR-301a/CXCR4	Inhibit apoptosis	ESCC	133
LncRNA LINC00511	microRNA-150-5p	Inhibit apoptosis	ESCC	134
LncRNA LINC00857	STAT3, MET	Inhibit apoptosis	EAC	135
LncRNA C9orF139	miR-661/HDAC11	Inhibit apoptosis	ESCC	136
LncRNA MIR205HG	miR-214/SOX4	Inhibit apoptosis	ESCC	137
LncRNA DLEU2	miR-30e-5p/E2F7	Inhibit apoptosis	ESCC	138
LncRNA DRAIC	miR-149-5p/NFIB	Inhibit autophagy	ESCC	189
LncRNA LINC00337	E2F4/TPX2	Induce autophagy	ESCC	190
LncRNA OIP5-AS1	GPX4	Inhibit ferroptosis	ESCC	236
LncRNA BBOX1-AS1	miR-513a-3p/SLC7A11	Inhibit ferroptosis	ESCC	237

**Table 7:** EC clinical trials targeting RCD modalities

Trial number	Drug/treatment	Number of included patients	Function	Phase
NCT00319735	Cetuximab, radiotherapy	41	Induce apoptosis	Phase 2
NCT00130689	Cetuximab	43	Induce apoptosis	Phase 2
NCT00735826	Vorinostat	10	Induce apoptosis	NA
NCT00037817	Decitabine/Depsipeptide	34	Induce apoptosis	Phase 1

## Abbreviations

3-MA: 3-methyladenine
ACD: Accidental cell death
AKT: Protein kinase B
ART: artemisinin
ATG: Autophagy-associated protein
ASC: Adaptor protein apoptosis-associated speck-like protein
AML: Acute myeloid leukemia
BCL-2: B-cell lymphoma-2
BAX: BCL-2-associated X protein
BAK1: BCL-2 homologous antagonist/killer 1
BID: BAX-like BH3 protein
BA: Betulinic acid
BSO: L-buthionine-sulfoximine
BH4: Tetrahydrobiopterin
cTNM: Clinical tumor enodeemetastasis
CT: Computed tomography
Ch‑19: 2,4,6‑trimethoxy‑4'‑nitrochalcone
cIAP1: cellular inhibitor of apoptosis 1
cIAP2: Cellular inhibitor of apoptosis 2
CPP: Periplocin
CMSP: P-hydroxylcinnamaldehyde
CPS-C: Capilliposide C
CUR: Curcumin
CQ: Chloroquine
Cys2: Cystine
Cys: Cysteine
CIS: Cisplatin
CoQ10: Coenzyme Q10
CoQ10H2: Ubiquinol-10
CTL: Cytotoxic T lymphocyte
CAR: Chimeric antigen receptor
CUR: Curcumin
DISC: Death-inducing signaling complex
DR: Death receptor
DSC: 3-deoxysappanchalcone
DVDMS: Sinoporphyrin sodium
DEX: Dexmedetomidine
DHA: Dihydroartemisinin
DPI: Diverse pharmacological inhibitor
DPP8/9: Dipeptidases 8/9
DAMPs: Damage-associated molecular patterns
EC: Esophageal cancer
EAC: Esophageal adenocarcinoma
ESCC: Esophageal squamous cell carcinoma
EUS: Endoscopic ultrasound
ESD: Endoscopic submucosal dissection
EMRL: Ligation-assisted endoscopic mucosal resection
EMRC: Cap-assisted endoscopic mucosal resection
EMSO: European Society for Medical Oncology
Ech: Echinatin
FLOT: 5-fluorouracile-leucovorine-oxaliplatine-docetaxel
FADD: Fas-associated protein with death domain
FINs: Ferroptosis inducers
FDA: the U.S. Food and Drug Administration
FSP1: Ferroptosis suppressor protein 1
GLP: Ganoderma lucidum polysaccharide
GSR: Glutathione reductase
GPX4: Glutathione peroxidase 4
Glu: Glutamate
GSS: Glutathione synthetase
GCL: Glutamate-cysteine ligase
GSH: Glutathione
GSSH: Oxidized glutathione
GCH1: GTP cyclohydrolase I
GSDM: Gasdermin
GZMA: Granzyme A
GZMB: Granzyme B
HCQ: Hydroxyquinoline
IARC: International Agency for Research on Cancer
IAPs: Inhibitor of apoptosis proteins
IKE: Imidazole ketone erastin
LBP: Lobaplatin
LPS: Lipopolysaccharide
LBP: Lobaplatin
LncRNAs: Long non-coding ribonucleic acids
LOX-1: Lectin-like oxidized low-density lipoprotein receptor-1
MCBS: Magnitude of Clinical Benefifit Score
MBM: Multiband mucosectomy
MIO: Minimally invasive esophagectomy
MCL-1: Myeloid cell leukemia-1
MOMP: Mitochondria outer membrane permeability
MAPK: mitogen-activated protein kinase
mTOR: Mammalian target of rapamycin
MDP: Muramyl dipeptide
MLKL: Mixed lineage kinase domain-like protein
NK: Natural killer
NOD2: Nucleotide binding oligomerization domain containing 2
NBT: Neobractatin
ORI: Oridonin
OPD': Ophiopogonin D'
PET: Positron emission tomography
PARAs: Proapoptotic receptor agonists
PDT: Photodynamic therapy
PPT: Picropodophyllotoxin
PI3K: Phosphatidylinositol 3-kinase
PE: Piperazine erastin
PUFA: Polyunsaturated fatty acid
PUFA-PLs: Polyunsaturated fatty acid phospholipids
PRRs: Cytosolic pattern recognition receptors
PAMPs: Pathogen-associated molecular patterns
PD-L1: Programmed death-ligand 1
QoL: Quality of life
Quina: Quinalizarin
RCD: Regulated cell death
RhTRAIL: Recombinant human TNF-related apoptosis-inducing ligand
ROS: Reactive oxygen species
Ro: Ginsenoside Ro
REA: Realgar
RIPK: Receptor-interacting protein kinase
SEMSs: Self-expanding metal stents
SMAC: Second mitochondrial-derived activators of caspases
SLC7A11: Solute carrier family 7 member 11
SCD1: Stearoyl-CoA desaturase
TRAIL: TNF-related apoptosis-inducing ligand
tBID: Truncated Bid
TF: Transferrin
TFR: Transferrin receptor
TNFα: Tumor necrosis factor alpha
TNFR: Tumor necrosis factor receptor
TRADD: Tumor necrosis factor receptor 1-associated death domain protein
TLR: Toll-like receptors
TRAF: Tumour necrosis factor receptor-associated factor
TRIF: TIR domain-containing adapter-inducing IFN-β
UA: Ursolic acid
ULK1: UNC-51-like kinase 1
VES: Vitamin E succinate
XIAP: X-linked inhibitor of apoptosis protein
ZBP1: Z-DNA-binding protein 1
